# Computationally Guided Structural Modification of Centaureidin: A Novel Approach for Enhancing Antioxidant and Antitumor Activities for Drug Development

**DOI:** 10.1111/cbdd.70149

**Published:** 2025-07-03

**Authors:** Reem S. Alruhaimi, Emadeldin M. Kamel, Sulaiman M. Alnasser, Ibrahim Elbagory, Ayman M. Mahmoud, Al Mokhtar Lamsabhi

**Affiliations:** ^1^ Department of Biology College of Science, Princess Nourah bint Abdulrahman University Riyadh Saudi Arabia; ^2^ Organic Chemistry Department Faculty of Science, Beni‐Suef University Beni‐Suef Egypt; ^3^ Department of Pharmacology and Toxicology College of Pharmacy, Qassim University Buraydah Saudi Arabia; ^4^ Department of Pharmaceutics Faculty of Pharmacy, Northern Border University Rafha Saudi Arabia; ^5^ Department of Life Sciences Faculty of Science and Engineering, Manchester Metropolitan University Manchester UK; ^6^ Departamento de Química Módulo 13, Universidad Autónoma de Madrid Madrid Spain; ^7^ Institute for Advanced Research in Chemical Sciences (IAdChem), Universidad Autónoma de Madrid Madrid Spain

**Keywords:** antioxidant, centaureidin, DFT, flavonoids, Mannich reaction

## Abstract

The development of novel therapeutic drugs with enhanced efficacy has gained significant attention in recent years. In this study, we aimed to enhance the radical scavenging and antitumor activities of centaureidin through computationally guided structural modifications. Centaureidin was initially isolated through extensive phytochemical fractionation from *Centaurea scoparia*. We employed Density Functional Theory (DFT) and multitarget molecular modeling to explore how modifying the carbon‐8 (C‐8) position influences bond dissociation enthalpies, radical scavenging mechanisms, and the structure‐antitumor activity relationships. Guided by computational analysis, we then modified the core skeleton of centaureidin using a facile multicomponent Mannich‐type synthesis, resulting in two newly substituted centaureidin analogues. The radical scavenging properties of centaureidin and its analogues CA1 and CA4 were investigated using DPPH and ABTS assays. CA1 and CA4 revealed more potent radical scavenging activities. In addition, both analogues were more effective in inhibiting the proliferation of the MCF‐7 cancer cell line. All tested compounds exhibited binding affinity towards caspase‐3 and the receptors EGFR, HER2 and VEGFR. In conclusion, structural modification of centaureidin resulted in enhanced antioxidant and cytotoxic activities. This comprehensive approach offers a streamlined and cost‐effective pathway for drug design and development, providing valuable insights for researchers in the field of therapeutic drug production.

## Graphical abstract

Computationally guided modifications of centaureidin led to the development of CA4, demonstrating enhanced antioxidant and antitumor activities. Molecular docking and in vitro assays confirm improved binding affinity and cytotoxicity, highlighting CA4's potential as a promising drug candidate for cancer therapy.

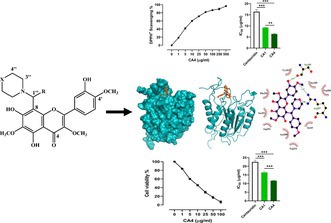

## 1 Introduction

Bioactive flavonoids, naturally occurring in edible plants, have been intensely researched for their potential applications in disease treatment, prevention, and drug design for several decades (Alruhaimi et al. 2023; Kamel et al. 2016; Sayed et al. 2020). The flavonoid centaureidin, a methylated variant (5,7,3′‐trihydroxy‐3,6,4′‐trimethoxyflavone), has been isolated from a variety of medicinal plants, including *Centaurea scoparia* (Ahmed and Kamel 2014), *Tanacetum microphyllum* (Abad et al. 1995), *Polymnia fruticose* (Beutler et al. 1993), and *Achillea clavennae* (Trifunović et al. 2006). It has previously been identified as having potent pharmacological effects, particularly cytotoxic and antioxidant activities (Ahmed and Kamel 2014; Beutler et al. 1993; Trifunović et al. 2006; Jachak et al. 2011). Yet, despite extensive discussions and inconsistencies dealing with the antioxidant and cytotoxic activities of flavonoids, there is a scarcity of reports on enhancing the activities of these compounds through computational guidance.

Chemotherapy of various tumors is marked by focusing on the functional activity of specific proteins and receptors such as caspase‐3, epidermal growth factor receptor (EGFR), human epidermal growth factor receptor 2 (HER2), and vascular endothelial growth factor receptor (VEGFR) (Mourad et al. 2021). Promoting apoptosis via caspase‐3 activation and targeting the active site domains of EGFR and HER2 along with the production of active tyrosine kinase inhibitors represent effective strategies for the chemotherapeutic treatment of many tumor cells (Harari 2004). Inhibition of EGFR promotes apoptosis in different cancer cells (Chae et al. 2011), and VEGFR is considered a critical signaling pro‐angiogenic factor responsible for angiogenesis in numerous malignancies (Fontanella et al. 2014; Le et al. 2020). Thus, anti‐tumors exhibiting activity against these receptors are clinically approved for the remedies of many tumors (Fontanella et al. 2014; Le et al. 2020).

The antioxidant activity of flavonoids could be attributed to the electronic structure, number, and position of hydroxyl groups (OH), and substitutions on the main skeleton (Havsteen 2002; Elsayed et al. 2020). The radical scavenging capability of flavonoids is essential in counteracting the harmful oxidative effects of reactive oxygen species (ROS). For a more thorough investigation, three mechanisms concerning the structure‐antioxidant activity relationship of flavonoids were extensively studied. These pathways include hydrogen atom transfer (HAT), single electron transfer followed by proton transfer (SET‐PT), and sequential proton loss electron transfer (SPLET) (Kamel et al. 2016; Elsayed et al. 2020). All three mechanisms can coexist in flavonoids, and their antioxidant action is believed to be promoted through a thermodynamic balance among their constituting parts (Ahmed and Kamel 2014). The HAT mechanism, for instance, involves calculating the bond dissociation enthalpy (BDE) of homolytic OH bond cleavage, while the SPLET pathway sums up the energy demand of two consecutive steps namely, heterolytic dissociation of the OH bond followed by an ionization of the anion. The SET‐PT pathway includes the ionization of the flavonoid followed by proton elimination (Leopoldini et al. 2004). These calculations target the constituting parameters for each path, thereby enabling an estimation of the structure‐antioxidant activity relationship of synthesized drugs. The Mannich reaction is a well‐known route for the production of therapeutic drugs such as β‐amino ketones and β‐amino aldehydes (Arend et al. 1998). This reaction is an efficient tool for introducing aminomethyl substituents into a specific position on the main skeleton of drugs, enhancing their biological activities. The structure of flavonoids can be altered by inserting one or two substituents into ring A through the combination of an aldehyde and secondary amine (Chen et al. 2006). Mannich bases have demonstrated wide‐ranging biological functions (Karthikeyan et al. 2006), which are primarily due to their ability to render target drugs soluble in polar solvents via biotransformation into ammonium salt (Karthikeyan et al. 2006). Therefore, this key reaction is valuable for the design of new candidate therapeutics to avoid drug resistance resulting from the continual administration of the same drugs.

In this study, we reported the synthesis of computationally guided novel centaureidin analogues (CAs) with improved cytotoxic efficacy. The proposed substituents to be inserted into the main skeleton of the flavonoid were determined according to the findings of DFT structure‐activity relationship calculations and multitargeted molecular docking analysis. Further, an in vitro cytotoxic assay of centaureidin and its modified analogues was performed to detect efficacy improvement.

## 2 Materials and Methods

### 2.1 General

^1^HNMR and ^13^CNMR spectra were recorded on Bruker AV‐500 NMR spectrometer (500 MHz and 125 MHz) using tetramethylsilane (TMS) as standard DMSO‐*d*_*6*_ NMR solvent. Chemical shifts (δ) are expressed in ppm and coupling constants (J) are presented in Hz. Shimadzu UV–vis 160i spectrophotometer was employed to measure UV spectra. HREIMS and EIMS mass spectra were recorded on a Finnigan MAT TSQ 700 mass spectrometer. IR spectra were obtained on KBr pellets on a Shimadzu FTIR‐8400 instrument. SANYO Gallenkamp instrument was used to measure the melting points. Silica gel 60 F_254_ Merck TLC plates were employed to carry out TLC analyses, while synthesized compounds were purified through chromatographing over columns using silica gel 60 (200–300 mesh) as a stationary phase. All commercially available starting materials and reagents employed in this investigation were purchased from Sigma Aldrich and Nasr Pharma. Reactions requiring an inert atmosphere were performed under Argon atmosphere in dry glassware with magnetic stirring. Chromatographic purifications were performed on the column using silica gel as a stationary phase.

### 2.2 Plant Material and Isolation of Centaureidin

*C. scoparia* aerial parts were collected from the eastern desert near Beni‐Suef city in May 2021. Plant identification was performed by staff members from the Botany and Microbiology Department at Beni‐Suef University. A voucher sample was kept in our natural product lab at Beni‐Suef University. Centaureidin was isolated from *C. scoparia* (3 kg) as previously reported by Ahmed and Kamel (2014) with slight modifications. The air‐dried powdered aerial parts of *C. scoparia* were exhaustively extracted by cold maceration in 70% ethanol (EtOH). The extraction solvent was then removed under reduced pressure to afford a dark brown sticky mass of the crude extract (455.8 g). The obtained residue underwent successive extraction using hexane, n‐butanol, and ethyl acetate (EtOAc). The EtOAc fraction (81 g) was subjected to fractionation over a polyamide 6S column eluted with an H_2_O/EtOH solvent system of decreasing polarity to collect a total of 65 subfractions. The obtained subfractions were combined into 19 subfractions based on their TLC profile (F1–F19). The collected subfractions were further dried at reduced pressure and subjected to an elaborate assessment using TLC and TDPC. Subfractions F14–F17 were combined and chromatographed over a polyamide column using methanol/H_2_O (8.5:1.5) to afford 12 major subfractions (C1–C12). The TLC behavior of C4–C8 revealed the presence of a major spot, and consequently, these subfractions were combined and purified over Sephadex LH‐20 columns eluted with methanol to yield centaureidin. The chemical structure of centaureidin was elucidated based on data from spectroscopic tools and by comparison with those previously reported (Long et al. 2003). The fractionation process was repeated until obtaining 374 mg of centaureidin as a starting material for our forthcoming investigations.

### 2.3 Synthesis of Centaureidin Analogues

**FIGURE 1:**
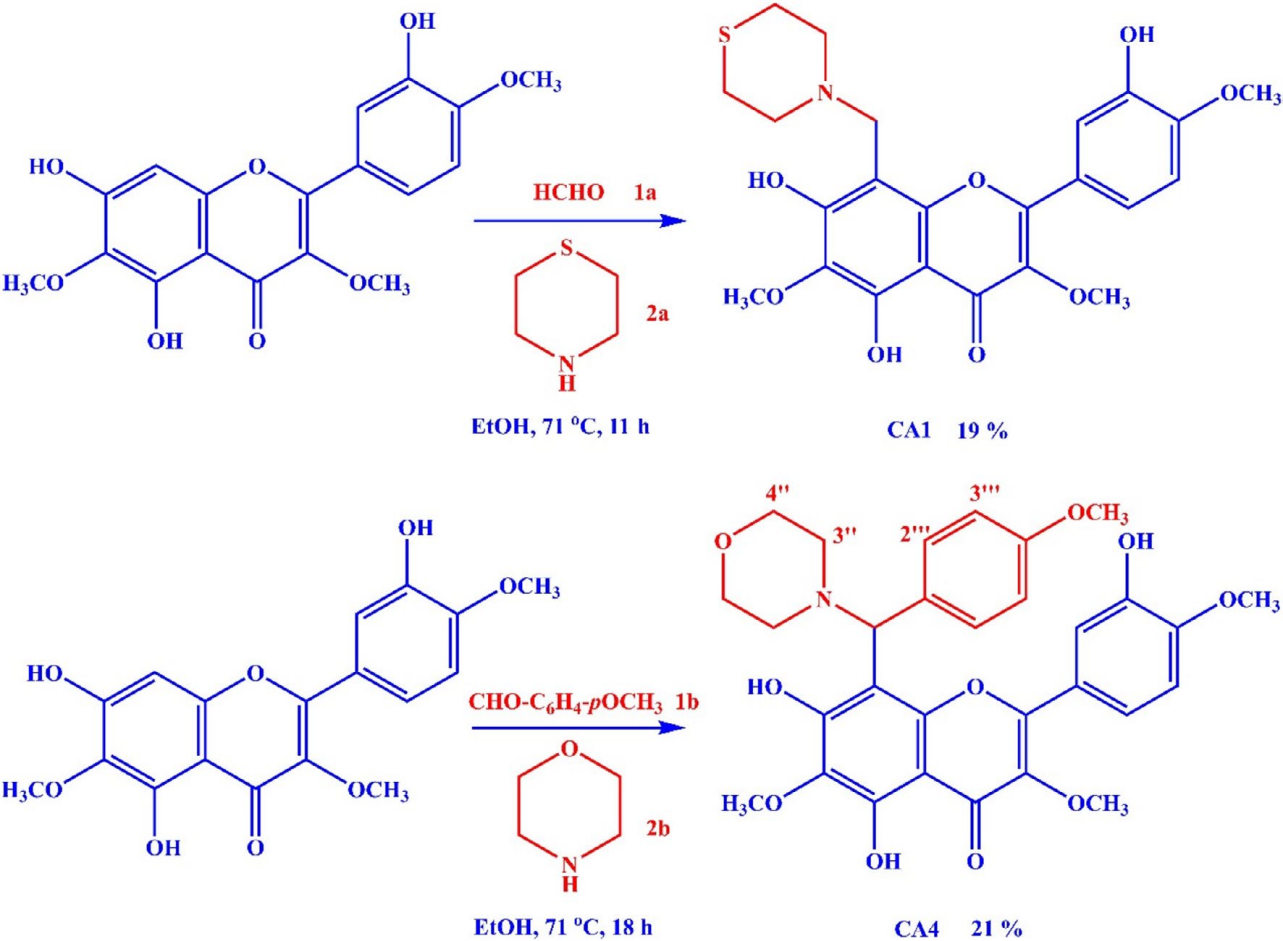
Three‐component Mannich reaction for the synthesis of CA1 and CA4 and the reaction building units, conditions, products, and yields.

To a solution of centaureidin (180 mg, 0.5 mmol) and the aldehyde 1a‐b (0.75 mmol) in EtOH (10 mL) at room temperature, the employed amine 2a‐b (0.75 mmol) was added dropwise while stirring. The reaction mixture was heated to 71°C while stirring for 11 and 18 h, respectively (Figure 1). By the end of the reaction, 25 mL of EtOAc and 25 mL of diluted hydrochloric acid (pH = 3) were poured into the reaction mixture. Subsequently, the aqueous layer was separated, and the solution was then neutralized to pH = 7 using 10% aqueous NaOH. The aqueous layer was then extracted with EtOAc three times (10 mL each). The combined organic layers were dried using anhydrous sodium sulfate, and the solvent was removed under reduced pressure. The obtained product was further purified by chromatographing over a silica gel column eluted by the solvent system hexane:EtOAc.

### 2.4 DFT Studies

DFT calculations executed were performed using the Gaussian 16 software package (Frisch et al. 2016). Geometrical structures of neutral flavonoids, radicals, anions, and cations were fully optimized at the B3LYP exchange‐correlation functional level without constraints (Lee et al. 1988; Becke 1988) by using the 6‐311G (d, p) basis set (Hehre and Radom 1986). At the same level of theory, frequency calculations were also performed to confirm that ground states show no imaginary frequency and to obtain zero‐point corrections. To provide a more complete characterization, the 6‐311++G (d, p) (Ditchfield et al. 1971) basis set was used for the single‐point energy calculations in the gas phase (*ɛ* = 1) and water solvent (*ɛ* = 78.4). Solvation effects were computed within the framework of the self‐consistent reaction field (SCRF) method using the polarizable continuum model (PCM) (Tomasi et al. 2005). Reaction enthalpies of the hydrogen radical (H˙), proton (H^+^), and electron (e−) were obtained as previously reported (Kamel et al. 2023).

### 2.5 Molecular Docking

A multitargeted molecular docking analysis was performed to figure out the binding interaction of centaureidin and its derivatives with the caspase‐3, EGFR, HER2, and VEGFR. The conformations of various drugs under investigation were fully optimized at the B3LYP exchange‐correlation functional level of theory without constraints (Lee et al. 1988; Becke 1988) using the 6‐311G (d, p) basis set (Hehre and Radom 1986). DFT studies were executed using Gaussian 16 software package (Frisch et al. 2016). The 3D crystal structures of target receptors were obtained from the Protein Data Bank (PDB) (1GFW, 2J6M, 3PP0, and 3B8Q for caspase‐3, and EGFR, HER2, and VEGFR2 kinase domains, respectively). The *in silico* molecular docking analysis was carried out using Autodock Tools (ADT) v1.5.6 and AutoDock Vina software (Trott and Olson 2010). The grid box sizes and dimensions are provided in Table S1. The targets were prepared using ADT v1.5.6. UCSF Chimera software was employed for eliminating drugs from the original PDB structure and for cleaning the original pdb generation (Pettersen et al. 2004). PyMOL v2.4 was utilized for high‐resolution image generation and binding site molecular visualizations.

### 2.6 In Vitro Radical‐Scavenging Activity

The scavenging activity of centaureidin, CA1, and CA4 was measured using DPPH and ABTS assays according to Brand‐Williams et al. (1995) and Re et al. (1999), respectively, using ascorbic acid as a standard.

### 2.7 Cytotoxicity Assay

MCF‐7 and HK‐2 cell lines (ATCC, Rockville, MD) were obtained from VACSERA (Egypt) and cultured in RPMI‐1640 supplemented with 10% fetal bovine serum, 1% L‐glutamine, HEPES buffer, and antibiotics, at 5% CO_2_ and 37°C. The cells were transferred to a 96‐well plate (1 × 10^4^ cells/well) and incubated for 24 h followed by treatment with different concentrations of centaureidin, CA1, CA4, and cisplatin for another 24 h. Cell viability was tested using the MTT assay in which 0.5 mg/mL MTT was added to the plate followed by incubation for 4 h at 37°C. DMSO (50 μL/well) was added, and the absorbance was measured at 590 nm after 10 min. The experiment was repeated 3 times (*N* = 3).

### 2.8 Statistical Analysis

Data of the IC_50_ were presented and mean ± SD, and the comparisons between groups were evaluated using one‐way ANOVA followed by Tukey's test on GraphPad Prism v8.

## 3 Results and Discussion

### 3.1 Computational Analysis of Synthesized Centaureidin Analogues

**FIGURE 2:**
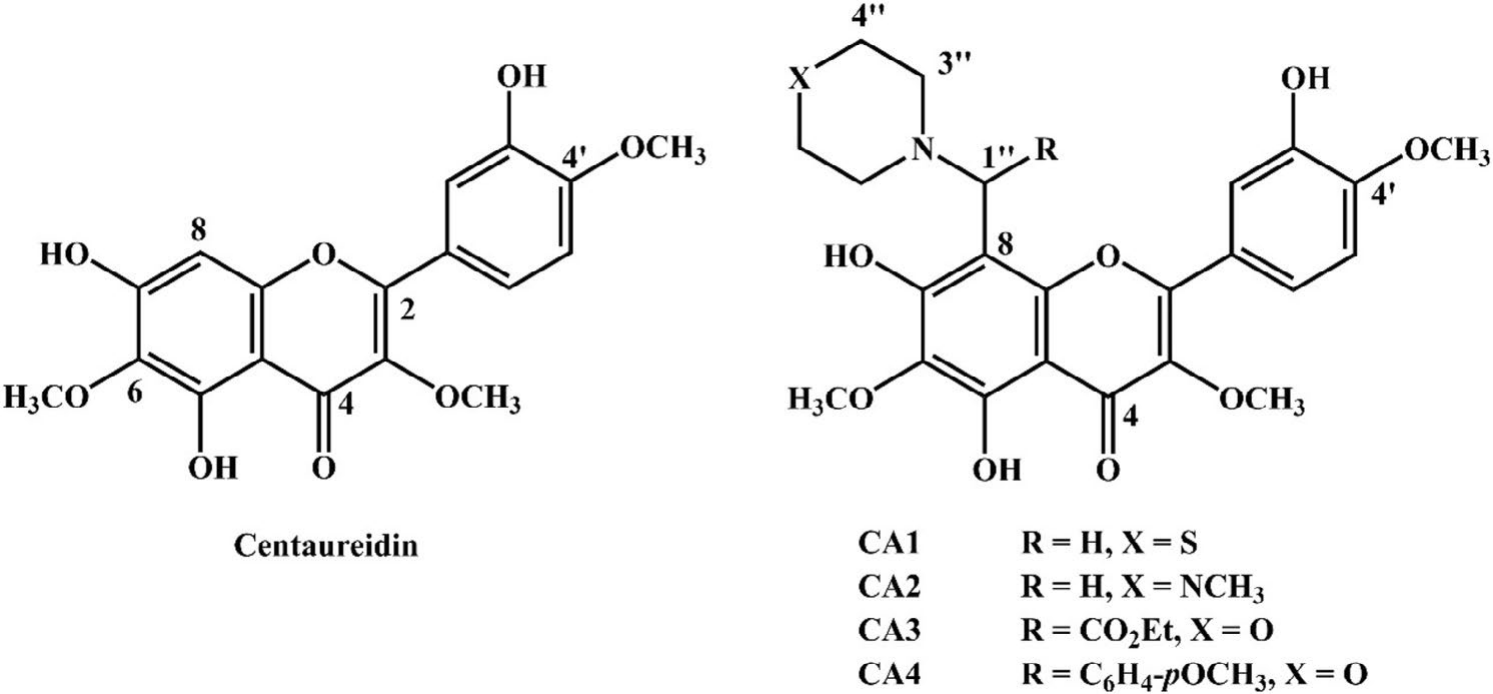
Chemical structures of centaureidin and four proposed centaureidin analogues.

Four proposed centaureidin analogues (CA1–CA4) (Figure 2) were investigated for their structure‐activity relationship by DFT calculations. These analogues were suggested based on information from extensive research on the Mannich‐type reaction of flavonoids. The influence of structural modification of centaureidin at C‐8 was studied by estimating the thermodynamic parameters responsible for the antioxidant mechanism of action of flavonoids, these parameters include BDE, proton affinity (PA), ionization potential (IP), proton dissociation enthalpy (PDE), and electron transfer enthalpy (ETE) (Ahmed and Kamel 2014; Elsayed et al. 2020; Kamel et al. 2023).

**FIGURE 3:**
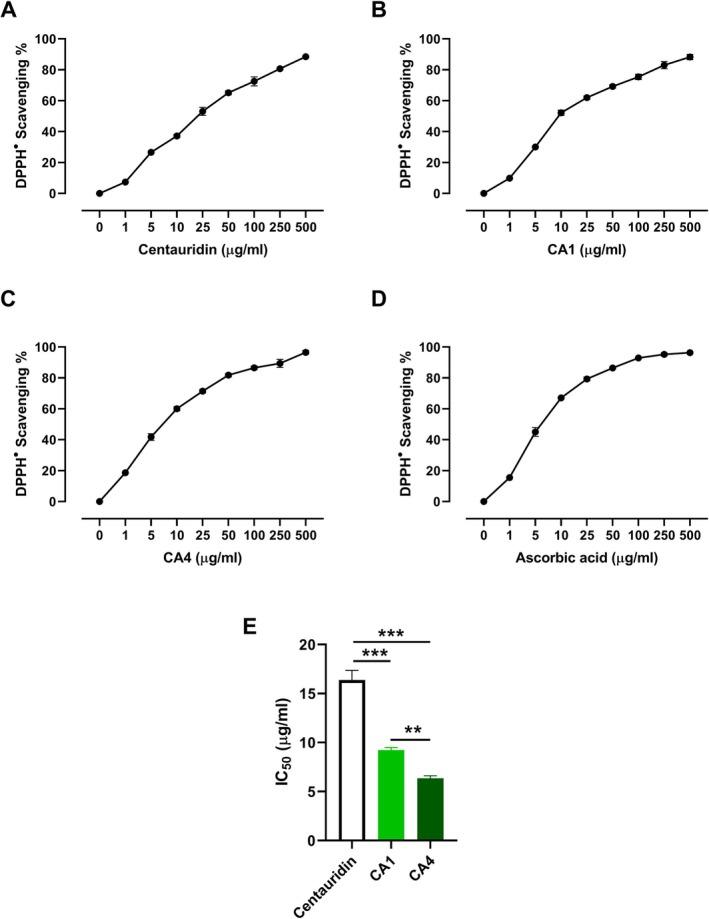
DPPH radical scavenging activity of centaureidin (A), CA1 (B), CA4 (C), and ascorbic acid (D), and (E) IC_50_ values. Data are mean ± SD, *N* = 3. **p < 0.01 and ***p < 0.001.

**FIGURE 4:**
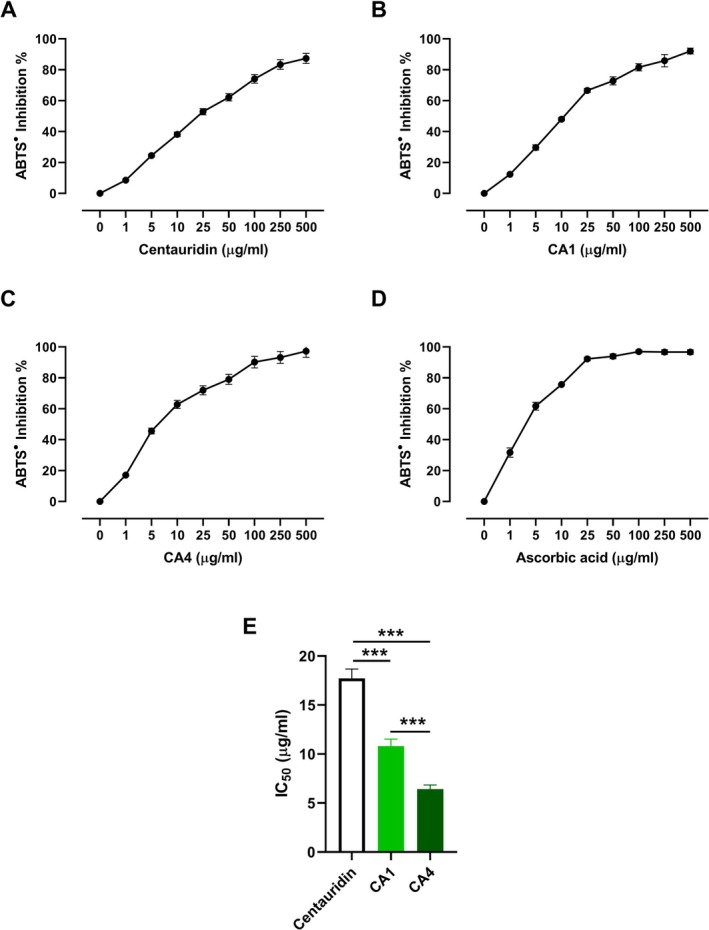
ABTS radical scavenging activity of centaureidin (A), CA1 (B), CA4 (C), and ascorbic acid (D), and (E) IC_50_ values. Data are mean ± SD, *N* = 3. ***p < 0.001.

**FIGURE 5:**
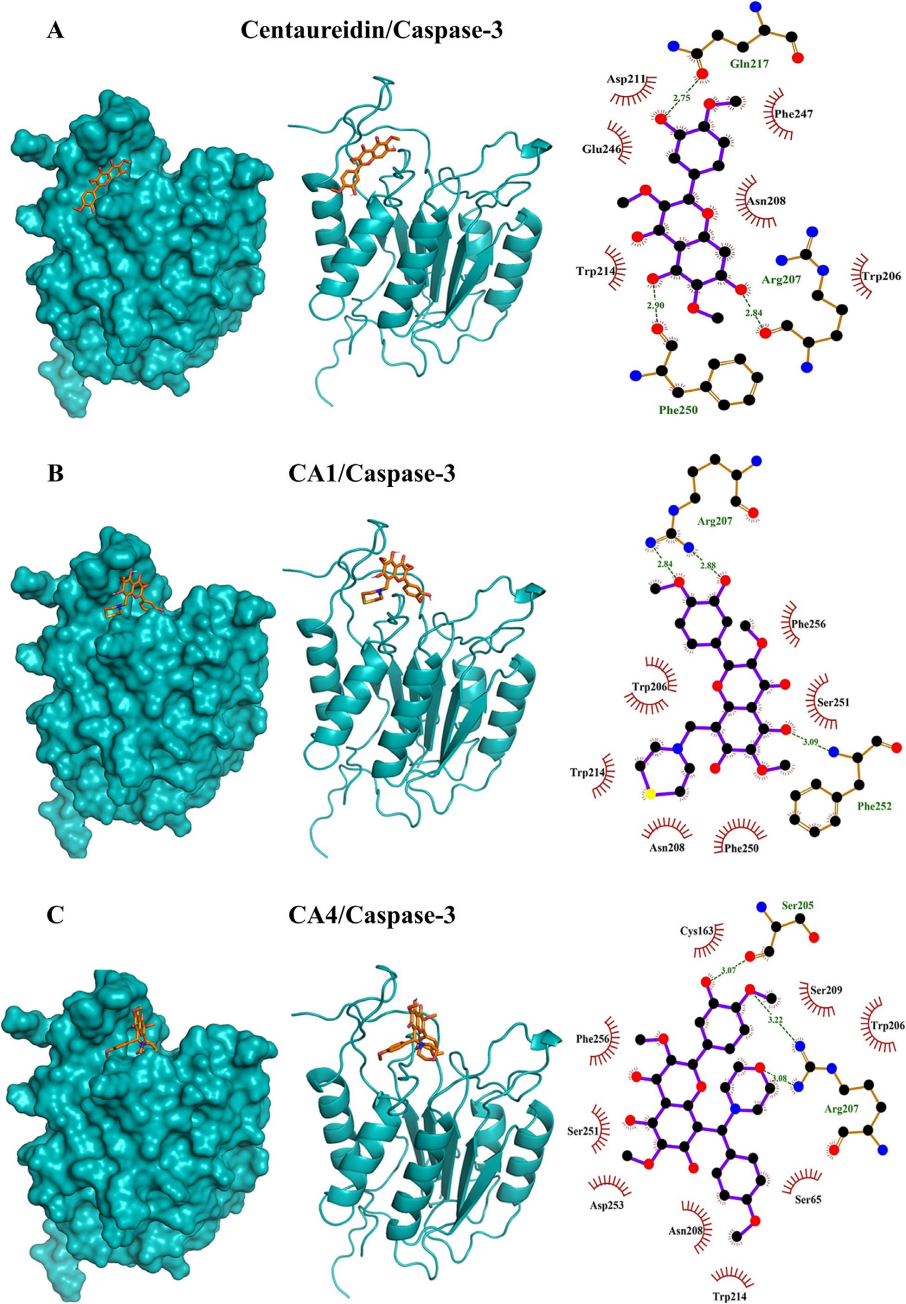
Molecular docking shows the binding affinity of centaureidin (A), CA1 (B), and CA4 (C) towards caspase‐3.

**FIGURE 6:**
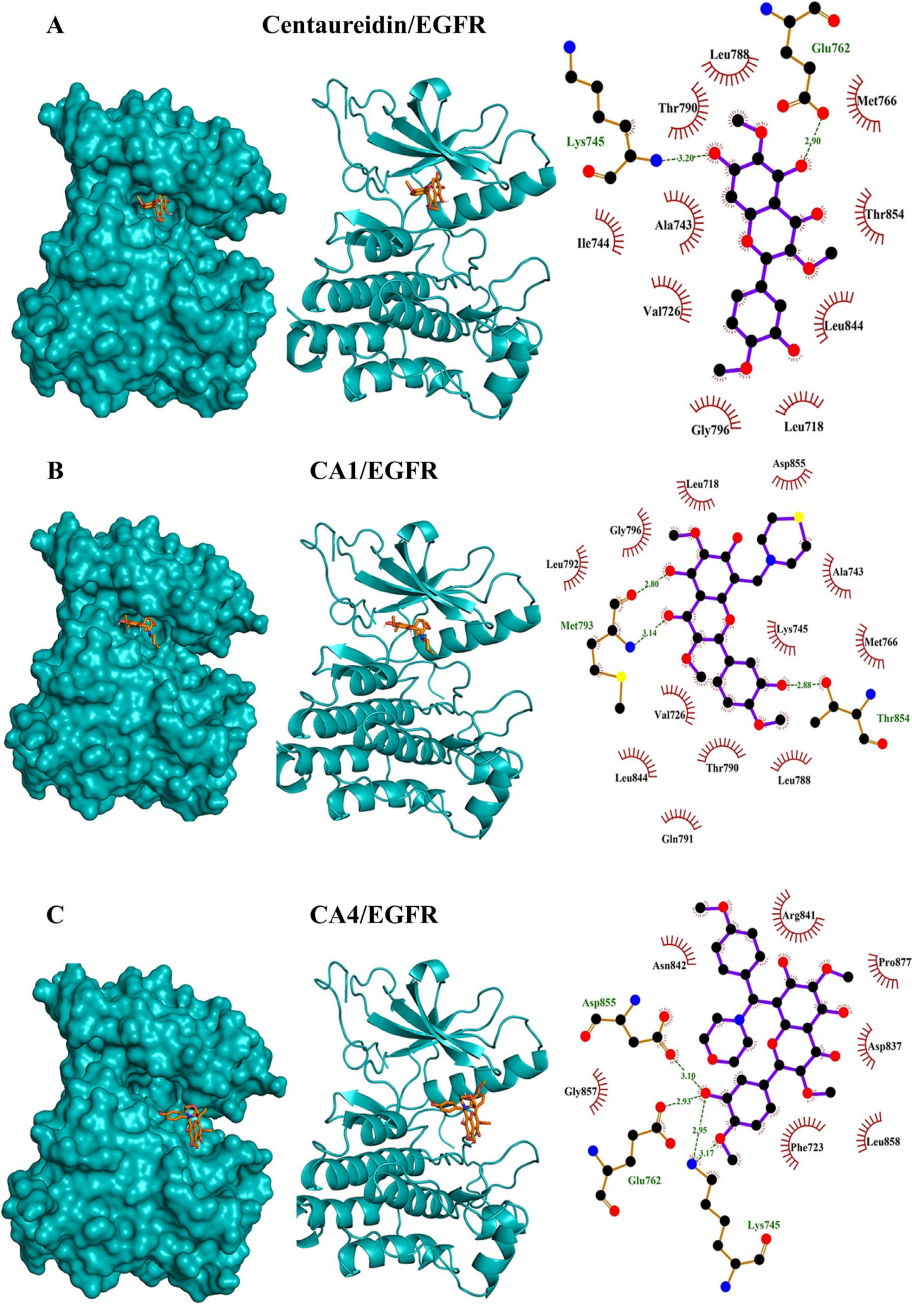
Molecular docking shows the binding affinity of centaureidin (A), CA1 (B), and CA4 (C) towards EGFR.

**FIGURE 7:**
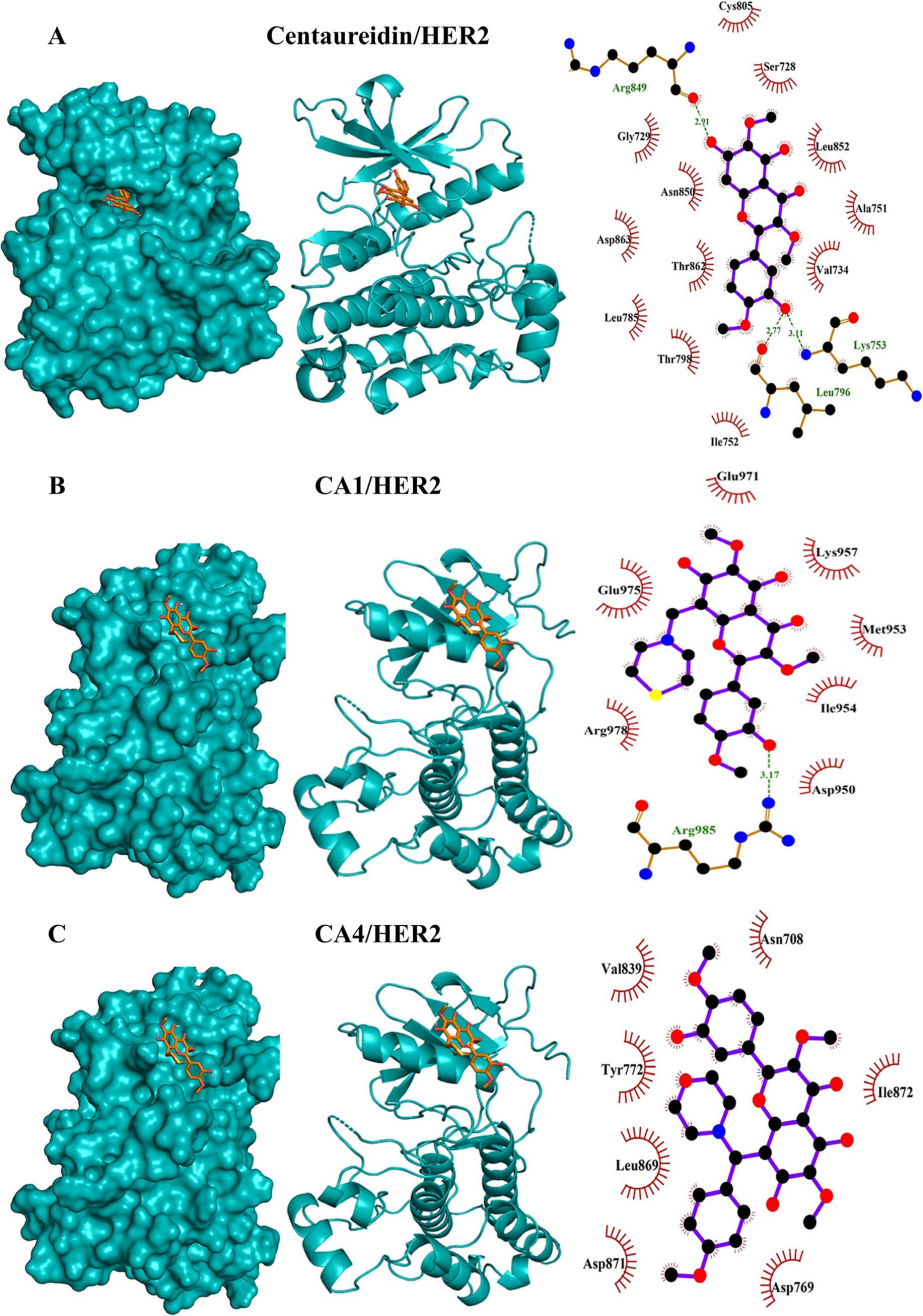
Molecular docking shows the binding affinity of centaureidin (A), CA1 (B), and CA4 (C) towards HER2.

**FIGURE 8:**
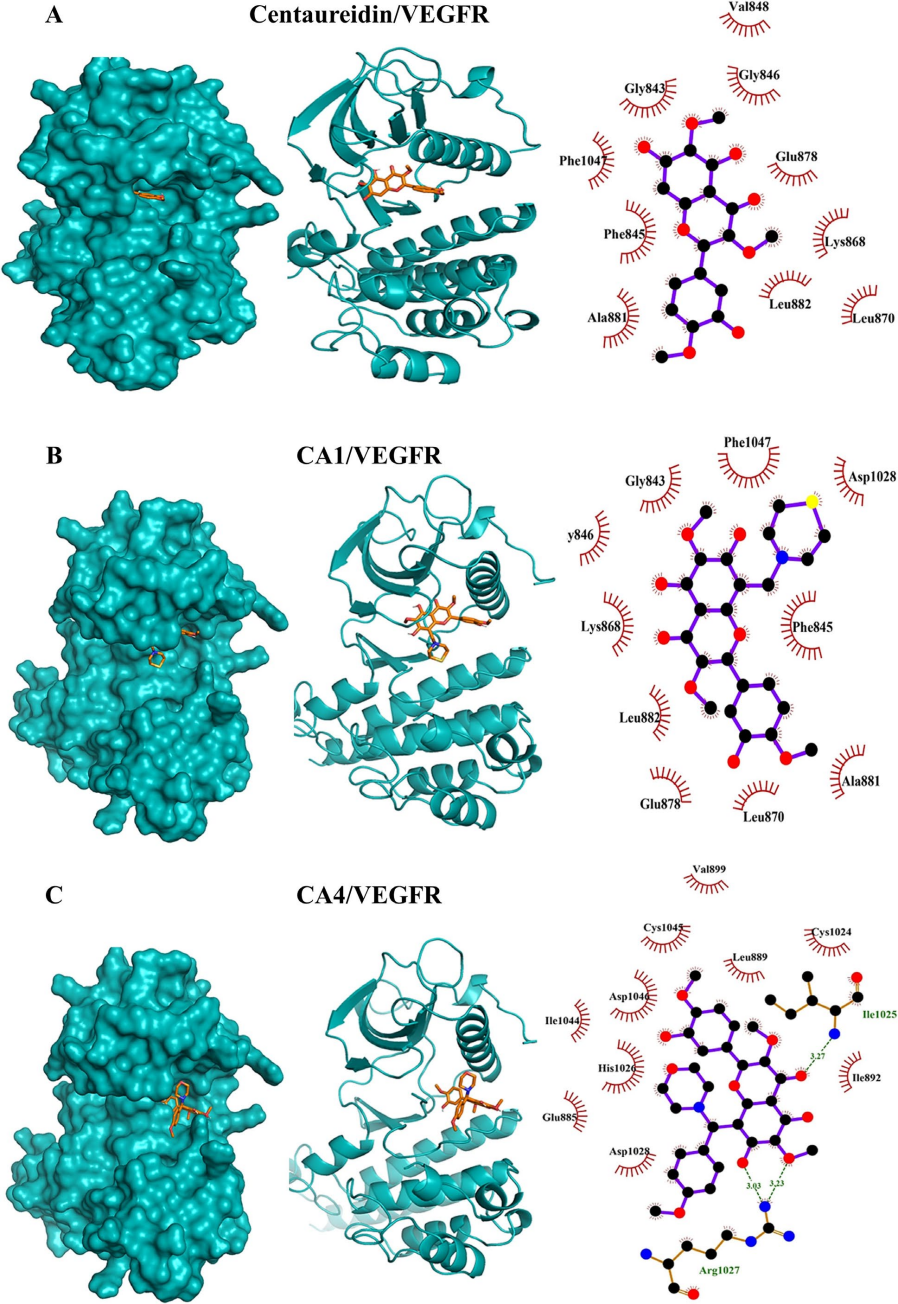
Molecular docking shows the binding affinity of centaureidin (A), CA1 (B), and CA4 (C) towards VEGFR.

**FIGURE 9:**
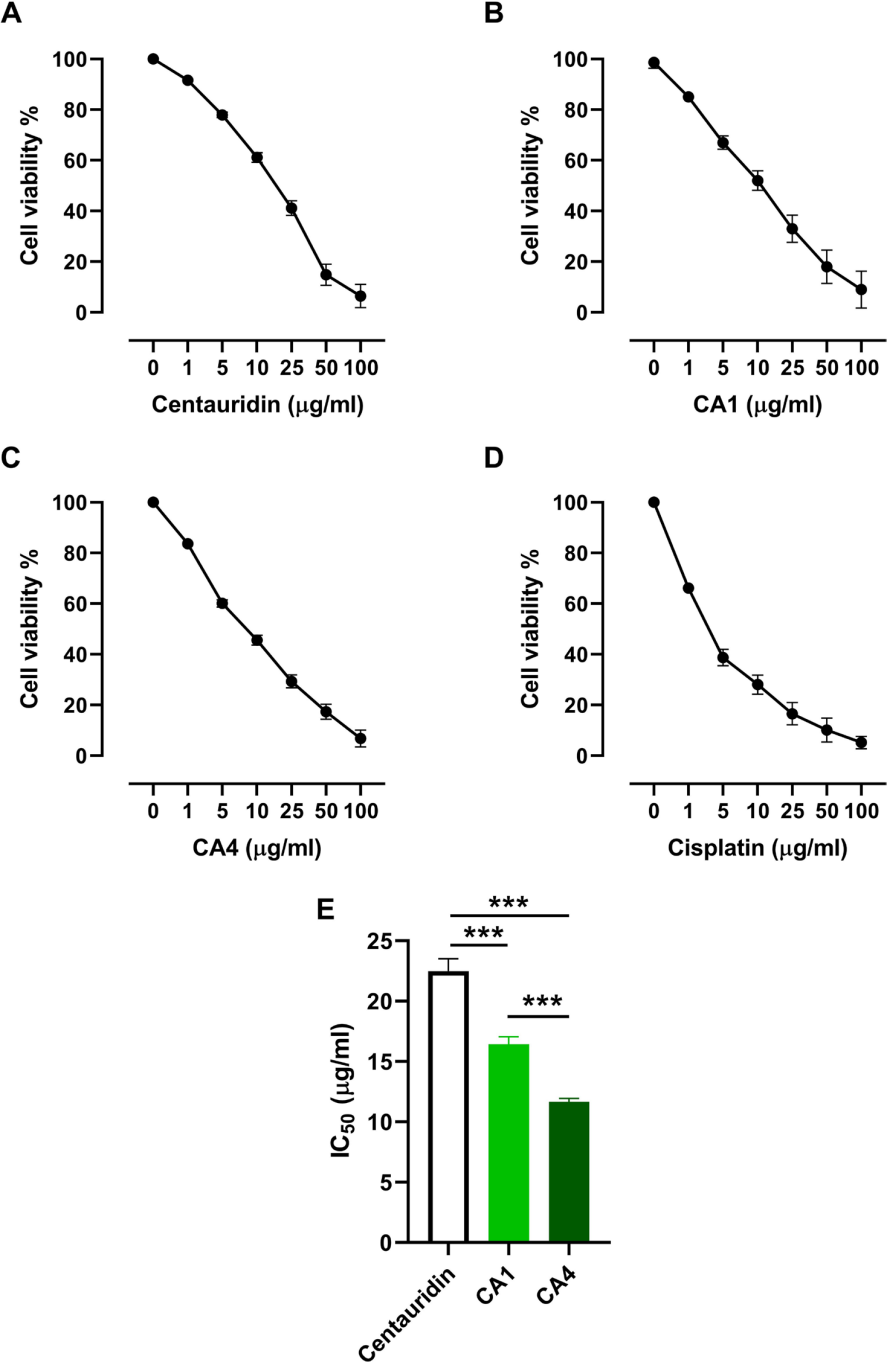
Antiproliferative activity of centaureidin (A), CA1 (B), CA4 (C), and cisplatin (D), and (E) IC_50_ values. Data are mean ± SD, *N* = 3. ***p < 0.001.

The results of DFT calculations of BDE values, radical scavenging thermodynamic parameters in a gaseous state, and water phase (kcal mol^−1^ at 298.15 K) for centaureidin and CAs for each minimum BDE OH (active site) are listed in Tables 1, 2, 3, respectively. Radicals formed by hydrogen abstraction from C‐3′ and C‐7 represent the most stable phenoxy radical in centaureidin and various CAs, respectively (Table 1). Consequently, positions 3′ and 7 are considered the main active site for radical scavenging action in centaureidin and the proposed analogues, respectively. The data in Table 2 indicated that the homolytic dissociation of the O‐H bond is the thermodynamically favored mechanism for the radical‐scavenging mechanism of action. The arrangement of BDE values of OH groups in the gaseous phase is the same as that estimated in the aqueous phase. The bulk polarity of water is similar to the cell environment in the gas phase. Therefore, our results focused on the mechanisms of antioxidant action in the water phase. The findings of our calculations in the water phase represented in Table 3 revealed that the SET‐PT is the thermodynamically disfavored pathway when compared with the SPLET route. This inference is attributed to the low energy demand of the initial step in the SPLET mechanism. Consequently, the SPLET pathway is considered to be the prevailing route for the radical scavenging activity of centaureidin and the proposed CAs.

**TABLE 1:** BDE values (in kcal/mol at 298.15 K) in the gas phase and water solution of various OH substituents of centaureidin and various CAs.

Flav	Phase	5‐OH	7‐OH	3′‐OH
1	Gas	77.92	71.63	68.98
	Water	75.32	69.38	67.55
CA1	Gas	93.28	83.19	83.39
	Water	91.21	82.64	82.78
CA2	Gas	82.57	82.36	83.17
	Water	82.15	81.66	81.82
CA3	Gas	90.50	82.77	82.93
	Water	88.31	80.96	81.15
CA4	Gas	91.17	81.90	83.70
	Water	90.92	81.13	82.75

Abbreviation: 1 = centaureidin.

**TABLE 2:** Reaction enthalpies (in kcal/mol) in the gaseous state of centaureidin and the proposed CAs.

Flav	Active site	SPLET			SET‐PT			BDE
		PA	ETE	PA + ETE	IP	PDE	IP + PDE	
1	3′‐OH	324.18	60.17	384.35	161.13	223.22	384.35	68.98
CA1	7‐OH	321.22	59.95	381.17	160.03	221.14	381.17	83.19
CA2	7‐OH	327.32	61.36	388.68	163.62	225.06	388.68	82.36
CA3	7‐OH	318.51	62.58	381.09	160.11	220.98	381.09	82.77
CA4	7‐OH	324.27	62.14	386.41	161.51	224.9	386.41	81.90

Abbreviation: 1 = centaureidin.

**TABLE 3:** Reaction enthalpies (in kcal/mol) in the water phase of centaureidin and the proposed CAs.

Flav	Active site	SPLET			SET‐PT			BDE
		PA	ETE	PA + ETE	IP	PDE	IP + PDE	
1	3′‐OH	29.09	37.83	66.92	68.89	−1.97	66.92	67.55
CA1	7‐OH	41.35	40.21	81.56	82.78	−1.22	81.56	82.64
CA2	7‐OH	40.98	40.59	81.57	83.31	−1.74	81.57	81.66
CA3	7‐OH	39.49	39.85	79.34	81.43	−2.09	79.34	80.96
CA4	7‐OH	40.46	39.17	79.63	80.94	−1.31	79.63	81.13

Abbreviations: 1 = centaureidin, ETE = electron transfer enthalpy, IP = ionization potential, PA = proton affinity, PDE = proton dissociation enthalpy.

Interestingly, modification of centaureidin with the proposed substituents resulted in increasing the BDE values of OH groups in all drugs by significant values (≈10–15 kcal/mol) and changing the active site from C‐3′ to C‐7. Also, the results of the computational analysis revealed that CA1 and CA4 possess the highest average BDE values for their constituting OH groups in both gas and water phases. In addition, CA1 and CA4 showed the highest PA values, which is the initial step in the thermodynamically prevailing radical‐scavenging mechanism in the water phase. These findings lead to the conclusion that CA1 and CA4 represent the most potent radical scavengers among the proposed analogues. Another leading factor for selecting CA4 is the presence of a chiral center (C‐1″) which might allow CA4 to interact with chiral macromolecules by modulating the absolute configuration that results in different pharmacodynamic influences because of the affinity variation among enantiomers. Also, it has been previously reported that the synthesis of flavonoids with a new stereogenic center through inserting amino acids was proven to be an effective strategy to produce novel antitumor drugs with improved potency (Pinto et al. 2021). Although CA1 and CA4 display higher overall BDE values, the 7‐OH group, identified as the primary active site, retains a BDE in aqueous solution that is within the functional range for hydrogen‐atom donation. Noteworthy, the antioxidant mechanisms such as HAT, SET‐PT, and SPLET can vary based on environmental factors, including solvent polarity, pH, and the nature of reactive species (Nakanishi et al. 2021; Apak et al. 2016). While our findings support SPLET as the dominant pathway in aqueous conditions, alternative mechanisms such as HAT may become more relevant in non‐polar or lipid‐rich environments.

Several studies have connected the antitumor activities of specific drugs to their ability to bind to DNA purine bases guanine and adenine at the N7 site (Chen et al. 2008). As represented in Tables 1, 2, 3, the BDE values of the proposed CAs were increased as a result of the structure modification. It has been shown that the decrease in BDE values leads to a disfavored binding of the drug to adenine at the N7 site (Chen et al. 2008). This influence is mainly attributed to the minimized capabilities of these OH groups (of low BDE) to form hydrogen bonds with the target receptors. These reasons also lead us to suggest CA1 and CA4 for synthesis and antitumor activity assay.

We selected CA1 and CA4 to be synthesized among the suggested centaureidin analogues due to their distinctive structural features and the promising results from our computational studies. CA1 was chosen because it contains sulfur, an element known for enhancing the pharmacological activity of compounds. Sulfur‐containing drugs are renowned for their diverse biological activities, including anti‐inflammatory, antimicrobial, and antitumor effects (Pathania et al. 2019; Barce Ferro et al. 2020). The presence of sulfur in CA1 is anticipated to improve its therapeutic efficacy. On the other hand, CA4 was selected due to the incorporation of a *p*‐methoxy group and a *p*‐C_6_H_5_‐OMe group in its structure. Methoxy groups are known to influence the electronic properties and increase the compound's lipophilicity, which can enhance its ability to interact with biological membranes and target proteins (Liew et al. 2020). The *p*‐C_6_H_5_‐OMe group is likely to further enhance these interactions through additional hydrophobic and π‐π stacking interactions (Triolo et al. 2020). Despite the high BDE values of CA1 and CA4, their enhanced activity can be attributed to their structural modifications, which improve their ability to stabilize radical intermediates and facilitate more effective HAT and single‐electron transfer processes. The sulfur in CA1 and the methoxy and *p*‐C_6_H_5_‐OMe groups in CA4 provide additional stabilization to the radical species, thereby enhancing the antioxidant efficacy of these analogues compared to the parent compound, centaureidin. The enhanced antioxidant activity observed for CA1 and CA4 could be attributed in part to the specific electronic and steric properties introduced by their respective substituents. In CA1, the thiomorpholine ring provides a highly polarizable sulfur atom that contributes to radical stabilization through n(S) → σ*C–O hyperconjugation and through‐bond π‐delocalization. Similarly, CA4 benefits from the +M (mesomeric) effect of the para‐methoxy and p‐methoxyphenyl substituents, which extend conjugation and facilitate spin delocalization over an expanded aromatic system. These effects, along with steric hindrance that may reduce radical–radical recombination, are summarized in Table S2. Additional computational evidence, including spin population analysis and HOMO–LUMO characteristics for the radical species, supports this interpretation. As shown in Table S3, the spin density on the key donor site (O7) is substantially reduced in both CA1 and CA4 compared to centaureidin, confirming more effective delocalization and stabilization of the radical intermediates.

### 3.2 Radical Scavenging Activity of Centaureidin, CA1, and CA4

The scavenging activities of centaureidin, CA1, and CA4 against DPPH (Figure 3) and ABTS (Figure 4) radicals were investigated in vitro. Centaureidin exhibited a concentration‐dependent DPPH (Figure 3A) and ABTS (Figure 4A) radical scavenging activities with reported IC_50_ values of 16.38 ± 0.97 μg/mL and 17.72 ± 0.89 μg/mL, respectively. CA1 showed a significantly higher radical scavenging activity towards DPPH (IC_50_ = 9.22 ± 0.27 μg/mL; Figure 3B) and ABTS (IC_50_ = 10.80 ± 0.72 μg/mL; Figure 4B) as compared to centaureidin (*p* < 0.001; Figures 3E and 4E). The scavenging activity of CA4 against DPPH (IC_50_ = 6.36 ± 0.24 μg/mL; Figure 3C) and ABTS (IC_50_ = 6.43 ± 0.42 μg/mL; Figure 4C) and its efficacy was significant when compared with either CA1 or centaureidin (Figures 3E and 4E). Therefore, both synthesized centaureidin analogues exhibited more potent DPPH radical scavenging activity than the parent compound. The DPPH assay is commonly used to investigate antioxidant activity; however, the ABTS assay is more reliable and accurate than DPPH (Floegel et al. 2011). We employed the ABTS assay and interestingly obtained the same outcomes, where CA4 showed more potent scavenging activity than CA1 and centaureidin. The superior activity of CA4 could be attributed to its optimized structural properties and binding interactions, as evidenced by both DFT calculations and molecular docking studies. DFT calculations revealed that CA4 possesses the highest average BDE values for its constituting OH groups in both gas and water phases, indicating its enhanced stability and efficacy in radical scavenging. Furthermore, CA4 showed the highest PA values, which is the initial step in the thermodynamically prevailing radical‐scavenging mechanism in the water phase. This superior antioxidant capacity is reflected in its more potent DPPH and ABTS radical scavenging activities compared to centaureidin and CA1.

### 3.3 Cytotoxic Activity of Centaureidin, CA1, and CA4

A multitargeted molecular docking analysis was executed to explore the binding interactions of centaureidin, CA1, and CA4 with caspase‐3, EGFR, HER2, and VEGFR (Figures 5, 6, 7, 8, respectively). In addition, the anti‐proliferative activity of centaureidin and its analogues on MCF‐7 was investigated in vitro (Figure 7). The binding energies, hydrogen bonding, and hydrophobic interactions of studied compounds against various targets are tabulated in Table S4. Centaureidin, CA1, and CA4 exhibited −7.0, −7.1, and −7.5 kcal/mol binding energies with caspase‐3, respectively. Although all studied drugs occupied the same binding site in the caspase‐3 structure, CA4 showed the lowest binding energy and a dense network of hydrophobic interactions. In addition, significant key residues (Ser205 and Arg207) were involved in the polar bonding in the active site of this complex (El Mansouri et al. 2023). As a result, CA4 activity as an antitumor could be mediated by the strong binding interaction to the caspase 3 binding cavity. The family of caspases involves aspartate‐specific cysteine proteases that controls apoptosis by acting as initiators and executioners (Kurokawa and Kornbluth 2009). In response to an apoptosis inducer/stimulus, a series of signaling activities involves the release of cytochrome *c* from the mitochondria into the cytosol occur (Kurokawa and Kornbluth 2009; Danial and Korsmeyer 2004). The released cytochrome *c* binds apoptotic protease activating factor‐1 and caspase‐9 to form apoptosome. Subsequently, the executioner caspase‐3 is activated and promote cell death via apoptosis by cleaving cellular proteins (Kurokawa and Kornbluth 2009).

In addition to their affinity to bind caspase‐3, molecular docking revealed the affinities of centaureidin, CA1, and CA4 towards HER2 (Figure 4) and VEGFR (Figure 5). This inference is attributed to the low binding energies and the high extent of polar and hydrophobic interactions. The reported binding energies with HER2 were −7.2, −7.1, and −7.7 kcal/mol for centaureidin, CA1, and CA4, respectively (Table S4). Centaureidin, CA1, and CA4 exhibited −7.6, −7.6, and −6.9 kcal/mol binding energies with VEGFR, respectively (Table S4). Among all studied compounds, only CA4 showed polar interactions with the residues Ile1025 and Arg1027 of VEGFR. Also, CA4 displayed the lowest binding energies with these two receptors. The high‐intensity interactions of CA4 with the proposed receptors contribute significantly to the affinity of CA4 to different active sites, molecular recognition, and orientation. The activation of HER receptors promote cell division (Tebbutt et al. 2013) and therefore represent an important target for the development of anti‐cancer agents. Ligand binding to the extracellular domain of HER receptors leads to dimerization of the receptor proteins and activation of downstream signaling. The activated signaling pathways inhibit apoptosis and promote cell proliferation (Tebbutt et al. 2013). Given the fundamental role of angiogenesis in tumor growth and progression, VEGFR has emerged as a key therapeutic target. Angiogenesis is the formation of new blood vessels essential for tumor cells to grow and proliferate. The newly formed blood vessels supply of oxygen and nutrients and provide the pathway for cancerous cells to metastasize (Yao and Zeng 2023). VEGF binding to its receptor VEGFR and the activation of downstream signaling regulate angiogenesis (Trimm and Red‐Horse 2023). EGF, which controls epithelial cells, and its receptor EGFR are over‐produced in tumors, including lung and brain cancers (Uribe et al. 2021). The high abundance of EGFR and its significant role in cell proliferation and tumorigenesis made it a popular target for the development of new therapies for cancer (Uribe et al. 2021). The results revealed that centaureidin, CA1, and CA4 exhibited −7.1, −7.2, and −7.8 kcal/mol binding energies with EGFR, respectively (Table S4). Contrary to centaureidin and CA1, CA4 was shown to occupy a different binding site with the target protein EGFR (Figure 6). CA4 exerted the highest binding affinity with EGFR (−6.9 kcal/mol). Also, CA4 is not deeply submerged in the binding pocket which might be attributed to its new structural features of CA4. CA1 displayed a relatively comparable pattern with centaureidin with a small degree of preference in favor of CA1. Thus, the results of our molecular docking analysis suggested a more potent activity of CA1 and CA4 against tumors by targeting caspase‐3 and different receptors.

CA4 contains a stereogenic center at the C‐1″ position, resulting from the Mannich‐type condensation, and was synthesized as a racemic mixture. Although no enantioselective synthesis or separation was performed in this study, the potential influence of stereochemistry on biological activity was considered. To explore possible stereoselective interactions with target proteins, we performed additional molecular docking simulations for both (R)‐ and (S)‐enantiomers of CA4 against caspase‐3, EGFR, HER2, and VEGFR2. The results indicated minimal differences in binding affinity (Δ ≤ 0.2 kcal/mol) and nearly identical binding poses, suggesting that stereochemistry at C‐1″ may not critically affect the primary binding interactions within these target sites. Nevertheless, we acknowledge that stereochemical differences could impact other aspects such as metabolic stability, cellular uptake, or protein selectivity in biological systems. Future work will involve the enantioselective synthesis and biological evaluation of CA4 enantiomers to assess potential enantiomer‐specific pharmacological profiles.

Many studies reported the in vitro antitumor activity of centaureidin against various cell lines (Csupor‐Löffler et al. 2009; Forgo et al. 2012); however, the exact mechanism of action is not fully understood. In addition to showing its binding affinity towards caspase‐3, HER2, VEGFR, and EGFR, our research aimed at improving the cytotoxic potential of centaureidin. Centaureidin showed a growth inhibitory activity against MCF‐7 cell line with IC_50_ value 22.49 ± 1.01 μg/mL (Figure 9A). CA1 showed a more potent cytotoxic effect on MCF‐7 (IC_50_ = 16.42 ± 0.63 μg/mL) (Figure 9B) and its effect was significant (*p* < 0.001) as compared to centaureidin (Figure 9E). The cytotoxic efficacy of CA4 was significantly higher than the anti‐proliferative activity of both centaureidin and CA1 (*p* < 0.001) and showed IC_50_ value of 11.65 ± 0.48 μg/mL (Figure 9C,E). Cisplatin was employed as a reference drug and showed IC_50_ value of 3.29 ± 0.12 μg/mL (Figure 9D). The higher cytotoxic efficacy of CA4 is likely to be attributed to the existence of a chiral center in its structure. Of note, all three compounds maintained cell viability of HK‐2 cells across a wide concentration range (0–1000 μg/mL), indicating no significant cytotoxic effects to normal cells (Figure S1). These in vitro findings align with the *in silico* data showing the more potent cytotoxic efficacy of CA4. Thus, this new compendious method for the production of novel therapeutic antitumor drugs offers an abridged and cost‐saving route for researchers in the area of drug design and development (Figure 9).

## 4 Conclusion

The synthesis of new antitumor drugs with improved efficacy represents a common challenge. In this investigation, we performed DFT studies and multitargeted molecular docking to explore the factors controlling the activity of four proposed centaureidin analogues as antioxidants and antitumor drugs. The outcomes of our DFT work shed light on various radical scavenging mechanisms of centaureidin analogues. Also, the results uncovered the promising efficacy of CA1 and CA4 as potent analogues of centaureidin. CA1 and CA4 were synthesized by three‐component Mannich reaction and both exhibited more potent radical scavenging and anti‐proliferative activities than centaureidin. Molecular docking revealed that CA1 and CA4 can bind to caspase‐3, EGFR, HER2, and VEGFR. Both compounds exhibited intensive polar and hydrophobic interactions suggesting the compatibility of these drugs to bind with the target proteins. This study provided a comprehensive approach that offers a streamlined and cost‐effective pathway for drug design and development.

## Conflicts of Interest

The authors declare no conflicts of interest.

## Supporting information


Data S1.

## Data Availability

The data that supports the findings of this study are available in the manuscript and Supporting Information of this article.
