# Supplementary material for: Computationally Guided Structural Modification of Centaureidin: A Novel Approach for Enhancing Antioxidant and Antitumor Activities for Drug Development
Source: Chem Biol Drug Des. 2025 Jul 3;106(1):e70149. doi: 10.1111/cbdd.70149 (PMC12231936; doi:10.1111/cbdd.70149)
Supplement: Supplementary file 1 — Data S1. [file CBDD-106-e70149-s001.docx]

Supplementary information

|  | Page |
| --- | --- |
| Cartesian coordinates of centaureidin, CA1, CA4, and all flavonoid radicals | 2 |
| Calculated thermodynamic parameters of centaureidin | 23 |
| Calculated thermodynamic parameters of CA1 | 23 |
| Calculated thermodynamic parameters of CA2 | 23 |
| Calculated thermodynamic parameters of CA3 | 23 |
| Calculated thermodynamic parameters of CA4 | 23 |
| ^1^H and ^13^C NMR spectral data of centaureidin, CA1 and CA4 | 24 |
| Spectral characterization of investigated compounds | 29 |
| Table S1. Molecular docking grid box size and dimensions | 31 |
| Table S2. Electronic & steric contributions of the new substituents | 31 |
| Table S3. Computational evidences | 32 |
| Table S4. Binding energies, polar bonds, and hydrophobic interactions of centaureidin, CA1, and CA4 with caspase-3, EGFR, HER2, and VEGFR2. | 32 |
| Figure S1. Effect of centaureidin, CA1, and CA4 on HK-2 cell viability. | 33 |

**Cartesian coordinates of centaureidin, CA1, CA4, and all flavonoid radicals**

**Centaureidin**

C -4.0945 -1.68819 0.00026

C -2.7217 -1.68819 0.00026

C -1.99976 -0.46307 0.00026

C -2.71763 0.7608 0.00063

C -4.13905 0.73142 0.00079

C -4.81055 -0.46655 0.0005

C -0.57803 -0.43353 0.

H -4.68629 1.68588 0.00087

C -0.62202 1.98559 0.00048

C 0.09399 0.7637 0.00002

C 0.16297 3.3105 0.00023

C 1.55805 3.29469 -0.00005

C -0.52068 4.5263 0.00017

C 2.26922 4.49446 0.00029

H 2.09673 2.33602 0.00077

C 0.19057 5.72652 -0.00049

H -1.62021 4.53894 0.0002

C 1.5853 5.7108 -0.00028

H 3.36883 4.48209 0.00071

O -1.99488 1.98552 0.00075

O 1.52358 0.79811 -0.00022

C 2.03322 -0.538 -0.00058

H 1.68925 -1.05116 0.87307

H 1.68884 -1.05085 -0.87424

H 3.10289 -0.51172 -0.00083

O 0.13332 -1.67404 0.00003

O -1.9877 -2.91544 -0.00003

H -2.59984 -3.65495 0.00006

O -6.24014 -0.50083 0.00063

H -6.54997 -0.9507 -0.78881

O -4.82345 -2.91844 0.00016

C -5.48321 -3.08248 1.25822

H -6.53486 -2.93093 1.13188

H -5.30713 -4.07135 1.62704

H -5.1013 -2.3679 1.95709

O -0.51067 6.97278 -0.00109

H 0.12078 7.69578 -0.01314

O 2.31475 6.94076 -0.00001

C 3.40947 6.84671 -0.91524

H 3.78366 7.82681 -1.12565

H 4.18716 6.25356 -0.48135

H 3.0767 6.38937 -1.82354

**Centaureidin-3′-O^•^**

C -4.0945 -1.68819 0.00026

C -2.7217 -1.68819 0.00026

C -1.99976 -0.46307 0.00026

C -2.71763 0.7608 0.00063

C -4.13905 0.73142 0.00079

C -4.81055 -0.46655 0.0005

C -0.57803 -0.43353 0.

H -4.68629 1.68588 0.00087

C -0.62202 1.98559 0.00048

C 0.09399 0.7637 0.00002

C 0.16297 3.3105 0.00023

C 1.55805 3.29469 -0.00005

C -0.52068 4.5263 0.00017

C 2.26922 4.49446 0.00029

H 2.09673 2.33602 0.00077

C 0.19057 5.72652 -0.00049

H -1.62021 4.53894 0.0002

C 1.5853 5.7108 -0.00028

H 3.36883 4.48209 0.00071

O -1.99488 1.98552 0.00075

O 1.52358 0.79811 -0.00022

C 2.03322 -0.538 -0.00058

H 1.68925 -1.05116 0.87307

H 1.68884 -1.05085 -0.87424

H 3.10289 -0.51172 -0.00083

O 0.13332 -1.67404 0.00003

O -1.9877 -2.91544 -0.00003

O -6.24014 -0.50083 0.00063

O -4.82345 -2.91844 0.00016

C -5.48321 -3.08248 1.25822

H -6.53486 -2.93093 1.13188

H -5.30713 -4.07135 1.62704

H -5.1013 -2.3679 1.95709

O -0.51067 6.97278 -0.00109

O 2.31475 6.94076 -0.00001

C 3.40947 6.84671 -0.91524

H 3.78366 7.82681 -1.12565

H 4.18716 6.25356 -0.48135

H 3.0767 6.38937 -1.82354

H -1.04667 -2.72549 0.00001

H -6.53835 -1.41333 0.00416

**Centaureidin-5-O^•^**

C -4.0945 -1.68819 0.00026

C -2.7217 -1.68819 0.00026

C -1.99976 -0.46307 0.00026

C -2.71763 0.7608 0.00063

C -4.13905 0.73142 0.00079

C -4.81055 -0.46655 0.0005

C -0.57803 -0.43353 0.

H -4.68629 1.68588 0.00087

C -0.62202 1.98559 0.00048

C 0.09399 0.7637 0.00002

C 0.16297 3.3105 0.00023

C 1.55805 3.29469 -0.00005

C -0.52068 4.5263 0.00017

C 2.26922 4.49446 0.00029

H 2.09673 2.33602 0.00077

C 0.19057 5.72652 -0.00049

H -1.62021 4.53894 0.0002

C 1.5853 5.7108 -0.00028

H 3.36883 4.48209 0.00071

O -1.99488 1.98552 0.00075

O 1.52358 0.79811 -0.00022

C 2.03322 -0.538 -0.00058

H 1.68925 -1.05116 0.87307

H 1.68884 -1.05085 -0.87424

H 3.10289 -0.51172 -0.00083

O 0.13332 -1.67404 0.00003

O -1.9877 -2.91544 -0.00003

O -6.24014 -0.50083 0.00063

H -6.54997 -0.9507 -0.78881

O -4.82345 -2.91844 0.00016

C -5.48321 -3.08248 1.25822

H -6.53486 -2.93093 1.13188

H -5.30713 -4.07135 1.62704

H -5.1013 -2.3679 1.95709

O -0.51067 6.97278 -0.00109

H 0.12078 7.69578 -0.01314

O 2.31475 6.94076 -0.00001

C 3.40947 6.84671 -0.91524

H 3.78366 7.82681 -1.12565

H 4.18716 6.25356 -0.48135

H 3.0767 6.38937 -1.82354

**Centaureidin-7-O^•^**

C -4.0945 -1.68819 0.00026

C -2.7217 -1.68819 0.00026

C -1.99976 -0.46307 0.00026

C -2.71763 0.7608 0.00063

C -4.13905 0.73142 0.00079

C -4.81055 -0.46655 0.0005

C -0.57803 -0.43353 0.

H -4.68629 1.68588 0.00087

C -0.62202 1.98559 0.00048

C 0.09399 0.7637 0.00002

C 0.16297 3.3105 0.00023

C 1.55805 3.29469 -0.00005

C -0.52068 4.5263 0.00017

C 2.26922 4.49446 0.00029

H 2.09673 2.33602 0.00077

C 0.19057 5.72652 -0.00049

H -1.62021 4.53894 0.0002

C 1.5853 5.7108 -0.00028

H 3.36883 4.48209 0.00071

O -1.99488 1.98552 0.00075

O 1.52358 0.79811 -0.00022

C 2.03322 -0.538 -0.00058

H 1.68925 -1.05116 0.87307

H 1.68884 -1.05085 -0.87424

H 3.10289 -0.51172 -0.00083

O 0.13332 -1.67404 0.00003

O -1.9877 -2.91544 -0.00003

O -6.24014 -0.50083 0.00063

O -4.82345 -2.91844 0.00016

C -5.48321 -3.08248 1.25822

H -6.53486 -2.93093 1.13188

H -5.30713 -4.07135 1.62704

H -5.1013 -2.3679 1.95709

O -0.51067 6.97278 -0.00109

H 0.12078 7.69578 -0.01314

O 2.31475 6.94076 -0.00001

C 3.40947 6.84671 -0.91524

H 3.78366 7.82681 -1.12565

H 4.18716 6.25356 -0.48135

H 3.0767 6.38937 -1.82354

H -1.04667 -2.72549 0.00001

**CA1**

C -4.0945 -1.68819 0.00026

C -2.7217 -1.68819 0.00026

C -1.99976 -0.46307 0.00026

C -2.71763 0.7608 0.00063

C -4.13905 0.73142 0.00079

C -4.81055 -0.46655 0.0005

C -0.57803 -0.43353 0.

C -0.62202 1.98559 0.00048

C 0.09399 0.7637 0.00002

C 0.16297 3.3105 0.00023

C 1.55805 3.29469 -0.00005

C -0.52068 4.5263 0.00017

C 2.26922 4.49446 0.00029

H 2.09673 2.33602 0.00077

C 0.19057 5.72652 -0.00049

H -1.62021 4.53894 0.0002

C 1.5853 5.7108 -0.00028

H 3.36883 4.48209 0.00071

O -1.99488 1.98552 0.00075

O 1.52358 0.79811 -0.00022

C 2.03322 -0.538 -0.00058

H 1.68925 -1.05116 0.87307

H 1.68884 -1.05085 -0.87424

H 3.10289 -0.51172 -0.00083

O 0.13332 -1.67404 0.00003

O -1.9877 -2.91544 -0.00003

O -6.24014 -0.50083 0.00063

O -4.82345 -2.91844 0.00016

C -5.48321 -3.08248 1.25822

H -6.53486 -2.93093 1.13188

H -5.30713 -4.07135 1.62704

H -5.1013 -2.3679 1.95709

O -0.51067 6.97278 -0.00109

O 2.31475 6.94076 -0.00001

C 3.40947 6.84671 -0.91524

H 3.78366 7.82681 -1.12565

H 4.18716 6.25356 -0.48135

H 3.0767 6.38937 -1.82354

H -1.04667 -2.72549 0.00001

H -6.53835 -1.41333 0.00416

C -4.90504 2.06741 0.0009

H -4.65246 2.62329 0.8796

H -4.63731 2.63212 -0.86762

N -6.35103 1.80317 -0.01297

C -7.02448 1.64493 -1.16899

C -7.05836 1.7091 1.12998

C -8.40671 1.39076 -1.23348

H -6.54579 0.84164 -1.68906

C -8.44219 1.45794 1.1683

H -6.90425 2.62796 1.65614

H -8.85102 2.17095 -1.81554

H -8.90265 2.26919 1.69246

H -6.85443 2.53319 -1.7408

H -8.54084 0.47083 -1.76323

H -8.59263 0.569 1.74454

H -6.59581 0.93639 1.7078

S -9.12371 1.29669 -0.03929

H 0.12121 7.69551 -0.00144

**CA1-3′-O^•^**

C -4.0945 -1.68819 0.00026

C -2.7217 -1.68819 0.00026

C -1.99976 -0.46307 0.00026

C -2.71763 0.7608 0.00063

C -4.13905 0.73142 0.00079

C -4.81055 -0.46655 0.0005

C -0.57803 -0.43353 0.

C -0.62202 1.98559 0.00048

C 0.09399 0.7637 0.00002

C 0.16297 3.3105 0.00023

C 1.55805 3.29469 -0.00005

C -0.52068 4.5263 0.00017

C 2.26922 4.49446 0.00029

H 2.09673 2.33602 0.00077

C 0.19057 5.72652 -0.00049

H -1.62021 4.53894 0.0002

C 1.5853 5.7108 -0.00028

H 3.36883 4.48209 0.00071

O -1.99488 1.98552 0.00075

O 1.52358 0.79811 -0.00022

C 2.03322 -0.538 -0.00058

H 1.68925 -1.05116 0.87307

H 1.68884 -1.05085 -0.87424

H 3.10289 -0.51172 -0.00083

O 0.13332 -1.67404 0.00003

O -1.9877 -2.91544 -0.00003

O -6.24014 -0.50083 0.00063

O -4.82345 -2.91844 0.00016

C -5.48321 -3.08248 1.25822

H -6.53486 -2.93093 1.13188

H -5.30713 -4.07135 1.62704

H -5.1013 -2.3679 1.95709

O -0.51067 6.97278 -0.00109

O 2.31475 6.94076 -0.00001

C 3.40947 6.84671 -0.91524

H 3.78366 7.82681 -1.12565

H 4.18716 6.25356 -0.48135

H 3.0767 6.38937 -1.82354

H -1.04667 -2.72549 0.00001

H -6.53835 -1.41333 0.00416

C -4.90504 2.06741 0.0009

H -4.65246 2.62329 0.8796

H -4.63731 2.63212 -0.86762

N -6.35103 1.80317 -0.01297

C -7.02448 1.64493 -1.16899

C -7.05836 1.7091 1.12998

C -8.40671 1.39076 -1.23348

H -6.54579 0.84164 -1.68906

C -8.44219 1.45794 1.1683

H -6.90425 2.62796 1.65614

H -8.85102 2.17095 -1.81554

H -8.90265 2.26919 1.69246

H -6.85443 2.53319 -1.7408

H -8.54084 0.47083 -1.76323

H -8.59263 0.569 1.74454

H -6.59581 0.93639 1.7078

S -9.12371 1.29669 -0.03929

**CA1-5-O^•^**

C -4.0945 -1.68819 0.00026

C -2.7217 -1.68819 0.00026

C -1.99976 -0.46307 0.00026

C -2.71763 0.7608 0.00063

C -4.13905 0.73142 0.00079

C -4.81055 -0.46655 0.0005

C -0.57803 -0.43353 0.

C -0.62202 1.98559 0.00048

C 0.09399 0.7637 0.00002

C 0.16297 3.3105 0.00023

C 1.55805 3.29469 -0.00005

C -0.52068 4.5263 0.00017

C 2.26922 4.49446 0.00029

H 2.09673 2.33602 0.00077

C 0.19057 5.72652 -0.00049

H -1.62021 4.53894 0.0002

C 1.5853 5.7108 -0.00028

H 3.36883 4.48209 0.00071

O -1.99488 1.98552 0.00075

O 1.52358 0.79811 -0.00022

C 2.03322 -0.538 -0.00058

H 1.68925 -1.05116 0.87307

H 1.68884 -1.05085 -0.87424

H 3.10289 -0.51172 -0.00083

O 0.13332 -1.67404 0.00003

O -1.9877 -2.91544 -0.00003

O -6.24014 -0.50083 0.00063

O -4.82345 -2.91844 0.00016

C -5.48321 -3.08248 1.25822

H -6.53486 -2.93093 1.13188

H -5.30713 -4.07135 1.62704

H -5.1013 -2.3679 1.95709

O -0.51067 6.97278 -0.00109

O 2.31475 6.94076 -0.00001

C 3.40947 6.84671 -0.91524

H 3.78366 7.82681 -1.12565

H 4.18716 6.25356 -0.48135

H 3.0767 6.38937 -1.82354

H -6.53835 -1.41333 0.00416

C -4.90504 2.06741 0.0009

H -4.65246 2.62329 0.8796

H -4.63731 2.63212 -0.86762

N -6.35103 1.80317 -0.01297

C -7.02448 1.64493 -1.16899

C -7.05836 1.7091 1.12998

C -8.40671 1.39076 -1.23348

H -6.54579 0.84164 -1.68906

C -8.44219 1.45794 1.1683

H -6.90425 2.62796 1.65614

H -8.85102 2.17095 -1.81554

H -8.90265 2.26919 1.69246

H -6.85443 2.53319 -1.7408

H -8.54084 0.47083 -1.76323

H -8.59263 0.569 1.74454

H -6.59581 0.93639 1.7078

S -9.12371 1.29669 -0.03929

H 0.12121 7.69551 -0.00144

**CA1-7-O^•^**

C -4.0945 -1.68819 0.00026

C -2.7217 -1.68819 0.00026

C -1.99976 -0.46307 0.00026

C -2.71763 0.7608 0.00063

C -4.13905 0.73142 0.00079

C -4.81055 -0.46655 0.0005

C -0.57803 -0.43353 0.

C -0.62202 1.98559 0.00048

C 0.09399 0.7637 0.00002

C 0.16297 3.3105 0.00023

C 1.55805 3.29469 -0.00005

C -0.52068 4.5263 0.00017

C 2.26922 4.49446 0.00029

H 2.09673 2.33602 0.00077

C 0.19057 5.72652 -0.00049

H -1.62021 4.53894 0.0002

C 1.5853 5.7108 -0.00028

H 3.36883 4.48209 0.00071

O -1.99488 1.98552 0.00075

O 1.52358 0.79811 -0.00022

C 2.03322 -0.538 -0.00058

H 1.68925 -1.05116 0.87307

H 1.68884 -1.05085 -0.87424

H 3.10289 -0.51172 -0.00083

O 0.13332 -1.67404 0.00003

O -1.9877 -2.91544 -0.00003

O -6.24014 -0.50083 0.00063

O -4.82345 -2.91844 0.00016

C -5.48321 -3.08248 1.25822

H -6.53486 -2.93093 1.13188

H -5.30713 -4.07135 1.62704

H -5.1013 -2.3679 1.95709

O -0.51067 6.97278 -0.00109

O 2.31475 6.94076 -0.00001

C 3.40947 6.84671 -0.91524

H 3.78366 7.82681 -1.12565

H 4.18716 6.25356 -0.48135

H 3.0767 6.38937 -1.82354

C -4.90504 2.06741 0.0009

H -4.65246 2.62329 0.8796

H -4.63731 2.63212 -0.86762

N -6.35103 1.80317 -0.01297

C -7.02448 1.64493 -1.16899

C -7.05836 1.7091 1.12998

C -8.40671 1.39076 -1.23348

H -6.54579 0.84164 -1.68906

C -8.44219 1.45794 1.1683

H -6.90425 2.62796 1.65614

H -8.85102 2.17095 -1.81554

H -8.90265 2.26919 1.69246

H -6.85443 2.53319 -1.7408

H -8.54084 0.47083 -1.76323

H -8.59263 0.569 1.74454

H -6.59581 0.93639 1.7078

S -9.12371 1.29669 -0.03929

H 0.12121 7.69551 -0.00144

H -1.04667 -2.72549 0.00001

**CA2**

C -4.0945 -1.68819 0.00026

C -2.7217 -1.68819 0.00026

C -1.99976 -0.46307 0.00026

C -2.71763 0.7608 0.00063

C -4.13905 0.73142 0.00079

C -4.81055 -0.46655 0.0005

C -0.57803 -0.43353 0.

C -0.62202 1.98559 0.00048

C 0.09399 0.7637 0.00002

C 0.16297 3.3105 0.00023

C 1.55805 3.29469 -0.00005

C -0.52068 4.5263 0.00017

C 2.26922 4.49446 0.00029

H 2.09673 2.33602 0.00077

C 0.19057 5.72652 -0.00049

H -1.62021 4.53894 0.0002

C 1.5853 5.7108 -0.00028

H 3.36883 4.48209 0.00071

O -1.99488 1.98552 0.00075

O 1.52358 0.79811 -0.00022

C 2.03322 -0.538 -0.00058

H 1.68925 -1.05116 0.87307

H 1.68884 -1.05085 -0.87424

H 3.10289 -0.51172 -0.00083

O 0.13332 -1.67404 0.00003

O -1.9877 -2.91544 -0.00003

O -6.24014 -0.50083 0.00063

O -4.82345 -2.91844 0.00016

C -5.48321 -3.08248 1.25822

H -6.53486 -2.93093 1.13188

H -5.30713 -4.07135 1.62704

H -5.1013 -2.3679 1.95709

O -0.51067 6.97278 -0.00109

O 2.31475 6.94076 -0.00001

C 3.40947 6.84671 -0.91524

H 3.78366 7.82681 -1.12565

H 4.18716 6.25356 -0.48135

H 3.0767 6.38937 -1.82354

C -4.90504 2.06741 0.0009

H -4.65246 2.62329 0.8796

H -4.63731 2.63212 -0.86762

N -6.35103 1.80317 -0.01297

C -7.02448 1.64493 -1.16899

C -7.05836 1.7091 1.12998

C -8.40671 1.39076 -1.23348

H -6.54579 0.84164 -1.68906

C -8.44219 1.45794 1.1683

H -6.90425 2.62796 1.65614

H -8.85102 2.17095 -1.81554

H -8.90265 2.26919 1.69246

H -6.85443 2.53319 -1.7408

H -8.54084 0.47083 -1.76323

H -8.59263 0.569 1.74454

H -6.59581 0.93639 1.7078

H 0.12121 7.69551 -0.00144

H -1.04667 -2.72549 0.00001

H -6.53835 -1.41333 0.00416

N -9.12371 1.29669 -0.03929

C -10.17371 2.32452 -0.08355

H -10.76782 2.18982 -0.96321

H -10.79497 2.23729 0.78324

H -9.72263 3.2946 -0.1029

**CA2-3′-O^•^**

C -4.0945 -1.68819 0.00026

C -2.7217 -1.68819 0.00026

C -1.99976 -0.46307 0.00026

C -2.71763 0.7608 0.00063

C -4.13905 0.73142 0.00079

C -4.81055 -0.46655 0.0005

C -0.57803 -0.43353 0.

C -0.62202 1.98559 0.00048

C 0.09399 0.7637 0.00002

C 0.16297 3.3105 0.00023

C 1.55805 3.29469 -0.00005

C -0.52068 4.5263 0.00017

C 2.26922 4.49446 0.00029

H 2.09673 2.33602 0.00077

C 0.19057 5.72652 -0.00049

H -1.62021 4.53894 0.0002

C 1.5853 5.7108 -0.00028

H 3.36883 4.48209 0.00071

O -1.99488 1.98552 0.00075

O 1.52358 0.79811 -0.00022

C 2.03322 -0.538 -0.00058

H 1.68925 -1.05116 0.87307

H 1.68884 -1.05085 -0.87424

H 3.10289 -0.51172 -0.00083

O 0.13332 -1.67404 0.00003

O -1.9877 -2.91544 -0.00003

O -6.24014 -0.50083 0.00063

O -4.82345 -2.91844 0.00016

C -5.48321 -3.08248 1.25822

H -6.53486 -2.93093 1.13188

H -5.30713 -4.07135 1.62704

H -5.1013 -2.3679 1.95709

O -0.51067 6.97278 -0.00109

O 2.31475 6.94076 -0.00001

C 3.40947 6.84671 -0.91524

H 3.78366 7.82681 -1.12565

H 4.18716 6.25356 -0.48135

H 3.0767 6.38937 -1.82354

C -4.90504 2.06741 0.0009

H -4.65246 2.62329 0.8796

H -4.63731 2.63212 -0.86762

N -6.35103 1.80317 -0.01297

C -7.02448 1.64493 -1.16899

C -7.05836 1.7091 1.12998

C -8.40671 1.39076 -1.23348

H -6.54579 0.84164 -1.68906

C -8.44219 1.45794 1.1683

H -6.90425 2.62796 1.65614

H -8.85102 2.17095 -1.81554

H -8.90265 2.26919 1.69246

H -6.85443 2.53319 -1.7408

H -8.54084 0.47083 -1.76323

H -8.59263 0.569 1.74454

H -6.59581 0.93639 1.7078

H -1.04667 -2.72549 0.00001

H -6.53835 -1.41333 0.00416

N -9.12371 1.29669 -0.03929

C -10.17371 2.32452 -0.08355

H -10.76782 2.18982 -0.96321

H -10.79497 2.23729 0.78324

H -9.72263 3.2946 -0.1029

**CA2-5-O^•^**

C -2.68009 2.55895 -0.10009

C -1.43967 2.9893 0.24057

C -0.34804 2.10824 0.20669

C -0.55119 0.76069 -0.1002

C -1.80948 0.33277 -0.51467

C -2.88776 1.22607 -0.48971

C 0.94051 2.57667 0.46425

C 1.80775 0.35399 0.04468

C 2.02369 1.70714 0.32408

C 3.01218 -0.55667 -0.21871

C 4.29886 -0.01495 -0.19062

C 2.81979 -1.92226 -0.47209

C 5.40172 -0.85116 -0.38297

H 4.43435 1.03465 -0.02173

C 3.88825 -2.73634 -0.65178

H 1.8267 -2.32751 -0.51808

C 5.19359 -2.22053 -0.59983

H 6.39322 -0.44772 -0.36286

O 0.49526 -0.20974 0.02088

O 3.36333 2.19117 0.45815

C 3.40202 3.25725 1.40681

H 2.75655 4.04756 1.08425

H 3.07592 2.89689 2.3602

H 4.40355 3.62486 1.4869

O 1.131 3.81333 0.8143

O -1.24517 4.34888 0.62982

O -4.19711 0.78774 -0.8547

O -3.78168 3.47426 -0.0611

C -3.85571 4.20823 -1.29232

H -3.97504 3.52802 -2.10995

H -4.69225 4.87545 -1.25924

H -2.95557 4.77226 -1.42563

O 3.68293 -4.13106 -0.89157

O 6.30484 -3.10091 -0.76484

C 7.41468 -2.61469 -0.00789

H 8.24135 -3.28676 -0.11469

H 7.69308 -1.64632 -0.36691

H 7.14039 -2.54793 1.02448

C -1.9733 -1.12488 -0.99104

H -1.84191 -1.16123 -2.0513

H -1.23055 -1.73367 -0.51864

N -3.30444 -1.64245 -0.644

C -3.48366 -1.6009 0.81618

C -3.41019 -3.03164 -1.11253

C -4.86667 -2.17393 1.18445

H -3.41497 -0.58703 1.15102

C -4.79062 -3.60289 -0.74311

H -2.64612 -3.62279 -0.64695

H -4.99213 -2.14546 2.24826

H -4.86034 -4.6158 -1.08374

H -2.72061 -2.18688 1.28567

H -5.63323 -1.59042 0.72065

H -5.55729 -3.01714 -1.20635

H -3.28563 -3.05802 -2.17625

N -4.95698 -3.56545 0.71542

C -3.90522 -4.37116 1.34581

H -4.03617 -4.36075 2.40569

H -3.9678 -5.37707 0.98614

H -2.94457 -3.96329 1.10077

H 4.41937 -4.63217 -0.53051

H -4.84795 1.29225 -0.35619

**CA2-7-O^•^**

C -4.0945 -1.68819 0.00026

C -2.7217 -1.68819 0.00026

C -1.99976 -0.46307 0.00026

C -2.71763 0.7608 0.00063

C -4.13905 0.73142 0.00079

C -4.81055 -0.46655 0.0005

C -0.57803 -0.43353 0.

C -0.62202 1.98559 0.00048

C 0.09399 0.7637 0.00002

C 0.16297 3.3105 0.00023

C 1.55805 3.29469 -0.00005

C -0.52068 4.5263 0.00017

C 2.26922 4.49446 0.00029

H 2.09673 2.33602 0.00077

C 0.19057 5.72652 -0.00049

H -1.62021 4.53894 0.0002

C 1.5853 5.7108 -0.00028

H 3.36883 4.48209 0.00071

O -1.99488 1.98552 0.00075

O 1.52358 0.79811 -0.00022

C 2.03322 -0.538 -0.00058

H 1.68925 -1.05116 0.87307

H 1.68884 -1.05085 -0.87424

H 3.10289 -0.51172 -0.00083

O 0.13332 -1.67404 0.00003

O -1.9877 -2.91544 -0.00003

O -6.24014 -0.50083 0.00063

O -4.82345 -2.91844 0.00016

C -5.48321 -3.08248 1.25822

H -6.53486 -2.93093 1.13188

H -5.30713 -4.07135 1.62704

H -5.1013 -2.3679 1.95709

O -0.51067 6.97278 -0.00109

O 2.31475 6.94076 -0.00001

C 3.40947 6.84671 -0.91524

H 3.78366 7.82681 -1.12565

H 4.18716 6.25356 -0.48135

H 3.0767 6.38937 -1.82354

C -4.90504 2.06741 0.0009

H -4.65246 2.62329 0.8796

H -4.63731 2.63212 -0.86762

N -6.35103 1.80317 -0.01297

C -7.02448 1.64493 -1.16899

C -7.05836 1.7091 1.12998

C -8.40671 1.39076 -1.23348

H -6.54579 0.84164 -1.68906

C -8.44219 1.45794 1.1683

H -6.90425 2.62796 1.65614

H -8.85102 2.17095 -1.81554

H -8.90265 2.26919 1.69246

H -6.85443 2.53319 -1.7408

H -8.54084 0.47083 -1.76323

H -8.59263 0.569 1.74454

H -6.59581 0.93639 1.7078

H -1.04667 -2.72549 0.00001

N -9.12371 1.29669 -0.03929

C -10.17371 2.32452 -0.08355

H -10.76782 2.18982 -0.96321

H -10.79497 2.23729 0.78324

H -9.72263 3.2946 -0.1029

H 0.12121 7.69551 -0.00144

**CA3**

C -4.0945 -1.68819 0.00026

C -2.7217 -1.68819 0.00026

C -1.99976 -0.46307 0.00026

C -2.71763 0.7608 0.00063

C -4.13905 0.73142 0.00079

C -4.81055 -0.46655 0.0005

C -0.57803 -0.43353 0.

C -0.62202 1.98559 0.00048

C 0.09399 0.7637 0.00002

C 0.16297 3.3105 0.00023

C 1.55805 3.29469 -0.00005

C -0.52068 4.5263 0.00017

C 2.26922 4.49446 0.00029

H 2.09673 2.33602 0.00077

C 0.19057 5.72652 -0.00049

H -1.62021 4.53894 0.0002

C 1.5853 5.7108 -0.00028

H 3.36883 4.48209 0.00071

O -1.99488 1.98552 0.00075

O 1.52358 0.79811 -0.00022

C 2.03322 -0.538 -0.00058

H 1.68925 -1.05116 0.87307

H 1.68884 -1.05085 -0.87424

H 3.10289 -0.51172 -0.00083

O 0.13332 -1.67404 0.00003

O -1.9877 -2.91544 -0.00003

O -6.24014 -0.50083 0.00063

O -4.82345 -2.91844 0.00016

C -5.48321 -3.08248 1.25822

H -6.53486 -2.93093 1.13188

H -5.30713 -4.07135 1.62704

H -5.1013 -2.3679 1.95709

O -0.51067 6.97278 -0.00109

O 2.31475 6.94076 -0.00001

C 3.40947 6.84671 -0.91524

H 3.78366 7.82681 -1.12565

H 4.18716 6.25356 -0.48135

H 3.0767 6.38937 -1.82354

C -4.90504 2.06741 0.0009

H -4.65246 2.62329 0.8796

N -6.35103 1.80317 -0.01297

C -7.02448 1.64493 -1.16899

C -7.05836 1.7091 1.12998

C -8.40671 1.39076 -1.23348

H -6.54579 0.84164 -1.68906

C -8.44219 1.45794 1.1683

H -6.90425 2.62796 1.65614

H -8.85102 2.17095 -1.81554

H -8.90265 2.26919 1.69246

H -6.85443 2.53319 -1.7408

H -8.54084 0.47083 -1.76323

H -8.59263 0.569 1.74454

H -6.59581 0.93639 1.7078

H 0.12121 7.69551 -0.00144

H -6.53835 -1.41333 0.00416

C -4.51972 2.88017 -1.24912

O -4.09227 2.50197 -2.33899

O -4.69662 4.2137 -1.07082

C -4.32872 4.93248 -2.25103

C -5.141 6.18686 -2.38321

H -4.48882 4.26324 -3.13109

H -3.23928 5.17166 -2.18835

H -4.85388 6.74868 -3.305

H -6.23039 5.94728 -2.44578

H -4.98087 6.85579 -1.50289

O -9.12371 1.29669 -0.03929

H -1.04667 -2.72549 0.00001

**CA3-3′-O^•^**

C -4.0945 -1.68819 0.00026

C -2.7217 -1.68819 0.00026

C -1.99976 -0.46307 0.00026

C -2.71763 0.7608 0.00063

C -4.13905 0.73142 0.00079

C -4.81055 -0.46655 0.0005

C -0.57803 -0.43353 0.

C -0.62202 1.98559 0.00048

C 0.09399 0.7637 0.00002

C 0.16297 3.3105 0.00023

C 1.55805 3.29469 -0.00005

C -0.52068 4.5263 0.00017

C 2.26922 4.49446 0.00029

H 2.09673 2.33602 0.00077

C 0.19057 5.72652 -0.00049

H -1.62021 4.53894 0.0002

C 1.5853 5.7108 -0.00028

H 3.36883 4.48209 0.00071

O -1.99488 1.98552 0.00075

O 1.52358 0.79811 -0.00022

C 2.03322 -0.538 -0.00058

H 1.68925 -1.05116 0.87307

H 1.68884 -1.05085 -0.87424

H 3.10289 -0.51172 -0.00083

O 0.13332 -1.67404 0.00003

O -1.9877 -2.91544 -0.00003

O -6.24014 -0.50083 0.00063

O -4.82345 -2.91844 0.00016

C -5.48321 -3.08248 1.25822

H -6.53486 -2.93093 1.13188

H -5.30713 -4.07135 1.62704

H -5.1013 -2.3679 1.95709

O -0.51067 6.97278 -0.00109

O 2.31475 6.94076 -0.00001

C 3.40947 6.84671 -0.91524

H 3.78366 7.82681 -1.12565

H 4.18716 6.25356 -0.48135

H 3.0767 6.38937 -1.82354

C -4.90504 2.06741 0.0009

H -4.65246 2.62329 0.8796

N -6.35103 1.80317 -0.01297

C -7.02448 1.64493 -1.16899

C -7.05836 1.7091 1.12998

C -8.40671 1.39076 -1.23348

H -6.54579 0.84164 -1.68906

C -8.44219 1.45794 1.1683

H -6.90425 2.62796 1.65614

H -8.85102 2.17095 -1.81554

H -8.90265 2.26919 1.69246

H -6.85443 2.53319 -1.7408

H -8.54084 0.47083 -1.76323

H -8.59263 0.569 1.74454

H -6.59581 0.93639 1.7078

H -6.53835 -1.41333 0.00416

C -4.51972 2.88017 -1.24912

O -4.09227 2.50197 -2.33899

O -4.69662 4.2137 -1.07082

C -4.32872 4.93248 -2.25103

C -5.141 6.18686 -2.38321

H -4.48882 4.26324 -3.13109

H -3.23928 5.17166 -2.18835

H -4.85388 6.74868 -3.305

H -6.23039 5.94728 -2.44578

H -4.98087 6.85579 -1.50289

O -9.12371 1.29669 -0.03929

H -1.04667 -2.72549 0.00001

**CA3-5-O^•^**

C -4.0945 -1.68819 0.00026

C -2.7217 -1.68819 0.00026

C -1.99976 -0.46307 0.00026

C -2.71763 0.7608 0.00063

C -4.13905 0.73142 0.00079

C -4.81055 -0.46655 0.0005

C -0.57803 -0.43353 0.

C -0.62202 1.98559 0.00048

C 0.09399 0.7637 0.00002

C 0.16297 3.3105 0.00023

C 1.55805 3.29469 -0.00005

C -0.52068 4.5263 0.00017

C 2.26922 4.49446 0.00029

H 2.09673 2.33602 0.00077

C 0.19057 5.72652 -0.00049

H -1.62021 4.53894 0.0002

C 1.5853 5.7108 -0.00028

H 3.36883 4.48209 0.00071

O -1.99488 1.98552 0.00075

O 1.52358 0.79811 -0.00022

C 2.03322 -0.538 -0.00058

H 1.68925 -1.05116 0.87307

H 1.68884 -1.05085 -0.87424

H 3.10289 -0.51172 -0.00083

O 0.13332 -1.67404 0.00003

O -1.9877 -2.91544 -0.00003

O -6.24014 -0.50083 0.00063

O -4.82345 -2.91844 0.00016

C -5.48321 -3.08248 1.25822

H -6.53486 -2.93093 1.13188

H -5.30713 -4.07135 1.62704

H -5.1013 -2.3679 1.95709

O -0.51067 6.97278 -0.00109

O 2.31475 6.94076 -0.00001

C 3.40947 6.84671 -0.91524

H 3.78366 7.82681 -1.12565

H 4.18716 6.25356 -0.48135

H 3.0767 6.38937 -1.82354

C -4.90504 2.06741 0.0009

H -4.65246 2.62329 0.8796

N -6.35103 1.80317 -0.01297

C -7.02448 1.64493 -1.16899

C -7.05836 1.7091 1.12998

C -8.40671 1.39076 -1.23348

H -6.54579 0.84164 -1.68906

C -8.44219 1.45794 1.1683

H -6.90425 2.62796 1.65614

H -8.85102 2.17095 -1.81554

H -8.90265 2.26919 1.69246

H -6.85443 2.53319 -1.7408

H -8.54084 0.47083 -1.76323

H -8.59263 0.569 1.74454

H -6.59581 0.93639 1.7078

H -6.53835 -1.41333 0.00416

C -4.51972 2.88017 -1.24912

O -4.09227 2.50197 -2.33899

O -4.69662 4.2137 -1.07082

C -4.32872 4.93248 -2.25103

C -5.141 6.18686 -2.38321

H -4.48882 4.26324 -3.13109

H -3.23928 5.17166 -2.18835

H -4.85388 6.74868 -3.305

H -6.23039 5.94728 -2.44578

H -4.98087 6.85579 -1.50289

O -9.12371 1.29669 -0.03929

H 0.12121 7.69551 -0.00144

**CA3-7-O^•^**

C -4.0945 -1.68819 0.00026

C -2.7217 -1.68819 0.00026

C -1.99976 -0.46307 0.00026

C -2.71763 0.7608 0.00063

C -4.13905 0.73142 0.00079

C -4.81055 -0.46655 0.0005

C -0.57803 -0.43353 0.

C -0.62202 1.98559 0.00048

C 0.09399 0.7637 0.00002

C 0.16297 3.3105 0.00023

C 1.55805 3.29469 -0.00005

C -0.52068 4.5263 0.00017

C 2.26922 4.49446 0.00029

H 2.09673 2.33602 0.00077

C 0.19057 5.72652 -0.00049

H -1.62021 4.53894 0.0002

C 1.5853 5.7108 -0.00028

H 3.36883 4.48209 0.00071

O -1.99488 1.98552 0.00075

O 1.52358 0.79811 -0.00022

C 2.03322 -0.538 -0.00058

H 1.68925 -1.05116 0.87307

H 1.68884 -1.05085 -0.87424

H 3.10289 -0.51172 -0.00083

O 0.13332 -1.67404 0.00003

O -1.9877 -2.91544 -0.00003

O -6.24014 -0.50083 0.00063

O -4.82345 -2.91844 0.00016

C -5.48321 -3.08248 1.25822

H -6.53486 -2.93093 1.13188

H -5.30713 -4.07135 1.62704

H -5.1013 -2.3679 1.95709

O -0.51067 6.97278 -0.00109

O 2.31475 6.94076 -0.00001

C 3.40947 6.84671 -0.91524

H 3.78366 7.82681 -1.12565

H 4.18716 6.25356 -0.48135

H 3.0767 6.38937 -1.82354

C -4.90504 2.06741 0.0009

H -4.65246 2.62329 0.8796

N -6.35103 1.80317 -0.01297

C -7.02448 1.64493 -1.16899

C -7.05836 1.7091 1.12998

C -8.40671 1.39076 -1.23348

H -6.54579 0.84164 -1.68906

C -8.44219 1.45794 1.1683

H -6.90425 2.62796 1.65614

H -8.85102 2.17095 -1.81554

H -8.90265 2.26919 1.69246

H -6.85443 2.53319 -1.7408

H -8.54084 0.47083 -1.76323

H -8.59263 0.569 1.74454

H -6.59581 0.93639 1.7078

C -4.51972 2.88017 -1.24912

O -4.09227 2.50197 -2.33899

O -4.69662 4.2137 -1.07082

C -4.32872 4.93248 -2.25103

C -5.141 6.18686 -2.38321

H -4.48882 4.26324 -3.13109

H -3.23928 5.17166 -2.18835

H -4.85388 6.74868 -3.305

H -6.23039 5.94728 -2.44578

H -4.98087 6.85579 -1.50289

O -9.12371 1.29669 -0.03929

H 0.12121 7.69551 -0.00144

H -1.04667 -2.72549 0.00001

**CA4**

C -4.0945 -1.68819 0.00026

C -2.7217 -1.68819 0.00026

C -1.99976 -0.46307 0.00026

C -2.71763 0.7608 0.00063

C -4.13905 0.73142 0.00079

C -4.81055 -0.46655 0.0005

C -0.57803 -0.43353 0.

C -0.62202 1.98559 0.00048

C 0.09399 0.7637 0.00002

C 0.16297 3.3105 0.00023

C 1.55805 3.29469 -0.00005

C -0.52068 4.5263 0.00017

C 2.26922 4.49446 0.00029

H 2.09673 2.33602 0.00077

C 0.19057 5.72652 -0.00049

H -1.62021 4.53894 0.0002

C 1.5853 5.7108 -0.00028

H 3.36883 4.48209 0.00071

O -1.99488 1.98552 0.00075

O 1.52358 0.79811 -0.00022

C 2.03322 -0.538 -0.00058

H 1.68925 -1.05116 0.87307

H 1.68884 -1.05085 -0.87424

H 3.10289 -0.51172 -0.00083

O 0.13332 -1.67404 0.00003

O -1.9877 -2.91544 -0.00003

O -6.24014 -0.50083 0.00063

O -4.82345 -2.91844 0.00016

C -5.48321 -3.08248 1.25822

H -6.53486 -2.93093 1.13188

H -5.30713 -4.07135 1.62704

H -5.1013 -2.3679 1.95709

O -0.51067 6.97278 -0.00109

O 2.31475 6.94076 -0.00001

C 3.40947 6.84671 -0.91524

H 3.78366 7.82681 -1.12565

H 4.18716 6.25356 -0.48135

H 3.0767 6.38937 -1.82354

C -4.90504 2.06741 0.0009

H -4.65246 2.62329 0.8796

N -6.35103 1.80317 -0.01297

C -7.02448 1.64493 -1.16899

C -7.05836 1.7091 1.12998

C -8.40671 1.39076 -1.23348

H -6.54579 0.84164 -1.68906

C -8.44219 1.45794 1.1683

H -6.90425 2.62796 1.65614

H -8.85102 2.17095 -1.81554

H -8.90265 2.26919 1.69246

H -6.85443 2.53319 -1.7408

H -8.54084 0.47083 -1.76323

H -8.59263 0.569 1.74454

H -6.59581 0.93639 1.7078

O -9.12371 1.29669 -0.03929

H 0.12121 7.69551 -0.00144

H -1.04667 -2.72549 0.00001

H -6.53835 -1.41333 0.00416

C -4.51972 2.88017 -1.24912

C -4.49966 4.27417 -1.1959

C -4.19067 2.22273 -2.43447

C -4.15127 5.0105 -2.328

H -4.75987 4.7922 -0.26146

C -3.84119 2.95911 -3.56673

H -4.20625 1.12404 -2.47654

C -3.82158 4.35279 -3.51373

H -4.13604 6.10928 -2.28631

H -3.5814 2.44046 -4.50107

O -3.46434 5.10802 -4.67429

C -4.6498 5.51151 -5.36479

H -4.38309 6.10227 -6.21612

H -5.18765 4.64435 -5.68675

H -5.26568 6.0898 -4.70815

**CA4-3′-O^•^**

C -4.0945 -1.68819 0.00026

C -2.7217 -1.68819 0.00026

C -1.99976 -0.46307 0.00026

C -2.71763 0.7608 0.00063

C -4.13905 0.73142 0.00079

C -4.81055 -0.46655 0.0005

C -0.57803 -0.43353 0.

C -0.62202 1.98559 0.00048

C 0.09399 0.7637 0.00002

C 0.16297 3.3105 0.00023

C 1.55805 3.29469 -0.00005

C -0.52068 4.5263 0.00017

C 2.26922 4.49446 0.00029

H 2.09673 2.33602 0.00077

C 0.19057 5.72652 -0.00049

H -1.62021 4.53894 0.0002

C 1.5853 5.7108 -0.00028

H 3.36883 4.48209 0.00071

O -1.99488 1.98552 0.00075

O 1.52358 0.79811 -0.00022

C 2.03322 -0.538 -0.00058

H 1.68925 -1.05116 0.87307

H 1.68884 -1.05085 -0.87424

H 3.10289 -0.51172 -0.00083

O 0.13332 -1.67404 0.00003

O -1.9877 -2.91544 -0.00003

O -6.24014 -0.50083 0.00063

O -4.82345 -2.91844 0.00016

C -5.48321 -3.08248 1.25822

H -6.53486 -2.93093 1.13188

H -5.30713 -4.07135 1.62704

H -5.1013 -2.3679 1.95709

O -0.51067 6.97278 -0.00109

O 2.31475 6.94076 -0.00001

C 3.40947 6.84671 -0.91524

H 3.78366 7.82681 -1.12565

H 4.18716 6.25356 -0.48135

H 3.0767 6.38937 -1.82354

C -4.90504 2.06741 0.0009

H -4.65246 2.62329 0.8796

N -6.35103 1.80317 -0.01297

C -7.02448 1.64493 -1.16899

C -7.05836 1.7091 1.12998

C -8.40671 1.39076 -1.23348

H -6.54579 0.84164 -1.68906

C -8.44219 1.45794 1.1683

H -6.90425 2.62796 1.65614

H -8.85102 2.17095 -1.81554

H -8.90265 2.26919 1.69246

H -6.85443 2.53319 -1.7408

H -8.54084 0.47083 -1.76323

H -8.59263 0.569 1.74454

H -6.59581 0.93639 1.7078

O -9.12371 1.29669 -0.03929

H -1.04667 -2.72549 0.00001

H -6.53835 -1.41333 0.00416

C -4.51972 2.88017 -1.24912

C -4.49966 4.27417 -1.1959

C -4.19067 2.22273 -2.43447

C -4.15127 5.0105 -2.328

H -4.75987 4.7922 -0.26146

C -3.84119 2.95911 -3.56673

H -4.20625 1.12404 -2.47654

C -3.82158 4.35279 -3.51373

H -4.13604 6.10928 -2.28631

H -3.5814 2.44046 -4.50107

O -3.46434 5.10802 -4.67429

C -4.6498 5.51151 -5.36479

H -4.38309 6.10227 -6.21612

H -5.18765 4.64435 -5.68675

H -5.26568 6.0898 -4.70815

**CA4-5-O^•^**

C -4.0945 -1.68819 0.00026

C -2.7217 -1.68819 0.00026

C -1.99976 -0.46307 0.00026

C -2.71763 0.7608 0.00063

C -4.13905 0.73142 0.00079

C -4.81055 -0.46655 0.0005

C -0.57803 -0.43353 0.

C -0.62202 1.98559 0.00048

C 0.09399 0.7637 0.00002

C 0.16297 3.3105 0.00023

C 1.55805 3.29469 -0.00005

C -0.52068 4.5263 0.00017

C 2.26922 4.49446 0.00029

H 2.09673 2.33602 0.00077

C 0.19057 5.72652 -0.00049

H -1.62021 4.53894 0.0002

C 1.5853 5.7108 -0.00028

H 3.36883 4.48209 0.00071

O -1.99488 1.98552 0.00075

O 1.52358 0.79811 -0.00022

C 2.03322 -0.538 -0.00058

H 1.68925 -1.05116 0.87307

H 1.68884 -1.05085 -0.87424

H 3.10289 -0.51172 -0.00083

O 0.13332 -1.67404 0.00003

O -1.9877 -2.91544 -0.00003

O -6.24014 -0.50083 0.00063

O -4.82345 -2.91844 0.00016

C -5.48321 -3.08248 1.25822

H -6.53486 -2.93093 1.13188

H -5.30713 -4.07135 1.62704

H -5.1013 -2.3679 1.95709

O -0.51067 6.97278 -0.00109

O 2.31475 6.94076 -0.00001

C 3.40947 6.84671 -0.91524

H 3.78366 7.82681 -1.12565

H 4.18716 6.25356 -0.48135

H 3.0767 6.38937 -1.82354

C -4.90504 2.06741 0.0009

H -4.65246 2.62329 0.8796

N -6.35103 1.80317 -0.01297

C -7.02448 1.64493 -1.16899

C -7.05836 1.7091 1.12998

C -8.40671 1.39076 -1.23348

H -6.54579 0.84164 -1.68906

C -8.44219 1.45794 1.1683

H -6.90425 2.62796 1.65614

H -8.85102 2.17095 -1.81554

H -8.90265 2.26919 1.69246

H -6.85443 2.53319 -1.7408

H -8.54084 0.47083 -1.76323

H -8.59263 0.569 1.74454

H -6.59581 0.93639 1.7078

O -9.12371 1.29669 -0.03929

H -6.53835 -1.41333 0.00416

C -4.51972 2.88017 -1.24912

C -4.49966 4.27417 -1.1959

C -4.19067 2.22273 -2.43447

C -4.15127 5.0105 -2.328

H -4.75987 4.7922 -0.26146

C -3.84119 2.95911 -3.56673

H -4.20625 1.12404 -2.47654

C -3.82158 4.35279 -3.51373

H -4.13604 6.10928 -2.28631

H -3.5814 2.44046 -4.50107

O -3.46434 5.10802 -4.67429

C -4.6498 5.51151 -5.36479

H -4.38309 6.10227 -6.21612

H -5.18765 4.64435 -5.68675

H -5.26568 6.0898 -4.70815

H 0.12121 7.69551 -0.00144

**CA4-7-O^•^**

C -4.0945 -1.68819 0.00026

C -2.7217 -1.68819 0.00026

C -1.99976 -0.46307 0.00026

C -2.71763 0.7608 0.00063

C -4.13905 0.73142 0.00079

C -4.81055 -0.46655 0.0005

C -0.57803 -0.43353 0.

C -0.62202 1.98559 0.00048

C 0.09399 0.7637 0.00002

C 0.16297 3.3105 0.00023

C 1.55805 3.29469 -0.00005

C -0.52068 4.5263 0.00017

C 2.26922 4.49446 0.00029

H 2.09673 2.33602 0.00077

C 0.19057 5.72652 -0.00049

H -1.62021 4.53894 0.0002

C 1.5853 5.7108 -0.00028

H 3.36883 4.48209 0.00071

O -1.99488 1.98552 0.00075

O 1.52358 0.79811 -0.00022

C 2.03322 -0.538 -0.00058

H 1.68925 -1.05116 0.87307

H 1.68884 -1.05085 -0.87424

H 3.10289 -0.51172 -0.00083

O 0.13332 -1.67404 0.00003

O -1.9877 -2.91544 -0.00003

O -6.24014 -0.50083 0.00063

O -4.82345 -2.91844 0.00016

C -5.48321 -3.08248 1.25822

H -6.53486 -2.93093 1.13188

H -5.30713 -4.07135 1.62704

H -5.1013 -2.3679 1.95709

O -0.51067 6.97278 -0.00109

O 2.31475 6.94076 -0.00001

C 3.40947 6.84671 -0.91524

H 3.78366 7.82681 -1.12565

H 4.18716 6.25356 -0.48135

H 3.0767 6.38937 -1.82354

C -4.90504 2.06741 0.0009

H -4.65246 2.62329 0.8796

N -6.35103 1.80317 -0.01297

C -7.02448 1.64493 -1.16899

C -7.05836 1.7091 1.12998

C -8.40671 1.39076 -1.23348

H -6.54579 0.84164 -1.68906

C -8.44219 1.45794 1.1683

H -6.90425 2.62796 1.65614

H -8.85102 2.17095 -1.81554

H -8.90265 2.26919 1.69246

H -6.85443 2.53319 -1.7408

H -8.54084 0.47083 -1.76323

H -8.59263 0.569 1.74454

H -6.59581 0.93639 1.7078

O -9.12371 1.29669 -0.03929

C -4.51972 2.88017 -1.24912

C -4.49966 4.27417 -1.1959

C -4.19067 2.22273 -2.43447

C -4.15127 5.0105 -2.328

H -4.75987 4.7922 -0.26146

C -3.84119 2.95911 -3.56673

H -4.20625 1.12404 -2.47654

C -3.82158 4.35279 -3.51373

H -4.13604 6.10928 -2.28631

H -3.5814 2.44046 -4.50107

O -3.46434 5.10802 -4.67429

C -4.6498 5.51151 -5.36479

H -4.38309 6.10227 -6.21612

H -5.18765 4.64435 -5.68675

H -5.26568 6.0898 -4.70815

H 0.12121 7.69551 -0.00144

H -1.04667 -2.72549 0.00001

**Calculated thermodynamic parameters of centaureidin.**

|  | Total Energy | ZPE | TC enthalpy | H (0 K) | BDE (0 K) | H (298 K) | BDE (298 K) |
| --- | --- | --- | --- | --- | --- | --- | --- |
| H• | -0.50215590 | 0 | 0.00236 | -0.50215590 |  | -0.49979590 |  |
| centaureidin | -1297.62518 | 0.317634 | 0.342677 | -1297.30754616 |  | -1297.28250316 |  |
|  | | | | | | | |
| 3'-O• | -1297.000661 | 0.305025 | 0.329474 | -1296.69563615 | 68.87 | -1296.67118715 | 69.98 |
| 5-O• | -1296.988029 | 0.304474 | 0.3295 | -1296.68355511 | 76.45 | -1296.65852911 | 77.92 |
| 7-O• | -1296.99795 | 0.304864 | 0.329386 | -1296.69308612 | 70.47 | -1296.66856412 | 71.63 |

**Calculated thermodynamic parameters of compound CA1.**

|  | Total Energy | ZPE | TC enthalpy | H (0 K) | BDE (0 K) | H (298 K) | BDE (298 K) |
| --- | --- | --- | --- | --- | --- | --- | --- |
| H• | -0.50215590 | 0 | 0.00236 | -0.50215590 |  | -0.49979590 |  |
| CA1 | -1946.609983 | 0.457635 | 0.490099 | -1946.15234797 |  | -1946.11988397 |  |
|  | | | | | | | |
| 3'-O• | -1945.964082 | 0.444632 | 0.47689 | -1945.51945015 | 82.04 | -1945.48719215 | 83.39 |
| 5-O• | -1945.948263 | 0.44403 | 0.476821 | -1945.50423336 | 91.59 | -1945.47144236 | 93.28 |
| 7-O• | -1945.964201 | 0.444339 | 0.476689 | -1945.51986175 | 81.78 | -1945.48751175 | 83.19 |

**Calculated thermodynamic parameters of compound CA2.**

|  | Total Energy | ZPE | TC enthalpy | H (0 K) | BDE (0 K) | H (298 K) | BDE (298 K) |
| --- | --- | --- | --- | --- | --- | --- | --- |
| H radical | -0.50215590 | 0 | 0.00236 | -0.50215590 |  | -0.49979590 |  |
| CA2 | -1643.061242 | 0.500986 | 0.534452 | -1642.56025551 |  | -1642.52678951 |  |
|  | | | | | | | |
| 3'-O• | -1642.41567 | 0.487952 | 0.521212 | -1641.92771802 | 81.82 | -1641.89445802 | 83.17 |
| 5-O• | -1642.416508 | 0.487464 | 0.521092 | -1641.92904381 | 80.98 | -1641.89541581 | 82.57 |
| 7-O• | -1642.417041 | 0.488145 | 0.521296 | -1641.92889609 | 81.08 | -1641.89574509 | 82.36 |

**Calculated thermodynamic parameters of compound CA3**

|  | Total Energy | ZPE | TC enthalpy | H (0 K) | BDE (0 K) | H (298 K) | BDE (298 K) |
| --- | --- | --- | --- | --- | --- | --- | --- |
| H• | -0.50215590 | 0 | 0.00236 | -0.50215590 |  | -0.49979590 |  |
| CA3 | -1890.876027 | 0.531614 | 0.569383 | -1890.34441325 |  | -1890.30664425 |  |
|  | | | | | | | |
| 3'-O• | -1890.230877 | 0.51862 | 0.556181 | -1889.71225713 | 81.58 | -1889.67469613 | 82.93 |
| 5-O• | -1890.218763 | 0.517997 | 0.556142 | -1889.70076626 | 88.79 | -1889.66262126 | 90.50 |
| 7-O• | -1890.231267 | 0.519023 | 0.556317 | -1889.71224423 | 81.58 | -1889.67495023 | 82.77 |

**Calculated thermodynamic parameters of compound CA4**

|  | Total Energy | ZPE | TC enthalpy | H (0 K) | BDE (0 K) | H (298 K) | BDE (298 K) |  |
| --- | --- | --- | --- | --- | --- | --- | --- | --- |
| H• | -0.50215590 | 0 | 0.00236 | -0.50215590 |  | -0.49979590 |  |  |
| CA4 | -1969.270428 | 0.573553 | 0.612773 | -1968.69687529 |  | -1968.65765529 |  |  |
|  | | | | | | | | |
| 3'-O• | -1968.624015 | 0.560323 | 0.599541 | -1968.06369207 | 82.22 | -1968.02447407 | 83.70 |  |
| 5-O• | -1968.612099 | 0.559723 | 0.599522 | -1968.05237612 | 89.32 | -1968.01257712 | 91.17 |  |
| 7-O• | -1968.626649 | 0.560414 | 0.5993 | -1968.06623546 | 80.62 | -1968.02734946 | 81.90 |  |

**^1^H and ^13^C NMR spectral data of centaureidin, CA1 and CA4**

**Centaureidin**: yellow oil, ^1^H NMR (500 MHz, DMSO-d6): δ (ppm) 7.66 (1H, d, J = 2.6 Hz, H-2'), 7.72 (1H, dd, J = 7.3, 2.6 Hz, H-6'), 6.91 (1H, d, J = 7.3 Hz, H-5'), 6.32 (1H, s, H-8), 3.95 (3H, s, 4'-OCH_3_), 3.79 (3H, s, 6-OCH_3_), 3.82 (3H, s, 3-OCH_3_); ^13^C NMR (125 MHz, DMSO-d6): δ (ppm) 179.15 (C-4), 157.24 (C-7), 156.78 (C-9), 155.81 (C-2), 154.79 (C-5), 149.64 (C-4'), 147.25 (C-3'), 139.27 (C-3), 131.43 (C-6), 123.52 (C-1'), 121.19 (C-6'), 116.11 (C-2'), 112.34 (C-5'), 105.98 (C-10), 95.37 (C-8), 61.17 (6-OCH_3_), 59.27 (3-OCH_3_), 57.91 (4'-OCH_3_). HREIMS mass spectrum revealed a [M + H] ^+^ ion at *m/z* 360.3124 corresponding to the molecular formula C_18_H_16_O_8_.

**Centaureidin**

**CA1**: yellow amorphous powder (45.17 mg, yield 19%) ^1^H NMR (500 MHz, DMSO-d6): δ (ppm) 7.69 (1H, d, J = 2.3 Hz, H-2'), 7.78 (1H, dd, J = 7.4, 2.3 Hz, H-6'), 6.89 (1H, d, J = 7.4 Hz, H-5'), 3.93 (3H, s, 4'-OCH_3_), 3.76 (3H, s, 6-OCH_3_), 3.88 (3H, s, 3-OCH_3_), 3.59 (2H, s, H-1''), 2.61 (2H, t, H-4''), 2.81 (2H, t, H-3''); ^13^C NMR (125 MHz, DMSO-d6): δ (ppm) 178.23 (C-4), 159.02 (C-2), 155.54 (C-7), 151.87 (C-9), 151.23 (C-5), 150.07 (C-4'), 146.90 (C-3'), 139.84 (C-3), 132.02 (C-6), 122.19 (C-1'), 120.57 (C-6'), 115.38 (C-2'), 112.75 (C-5'), 106.27 (C-8), 103.28 (C-10), 61.81 (6-OCH_3_), 60.08 (C-3''), 58.65 (3-OCH_3_), 55.98 (4'-OCH_3_), 51.23 (C-1''), 29.65 (C-4''). HREIMS (m/z): Calcd for C₂₃H₂₅NO₈S [M]⁺ 475.1301; found 475.1208.

**CA1**

**CA4**: yellowish-white amorphous powder (59.39 mg, yield 21%) , ^1^H NMR (500 MHz, DMSO-d6): δ (ppm) 7.63 (1H, d, J = 2.2 Hz, H-2'), 7.74 (1H, dd, J = 7.5, 2.2 Hz, H-6'), 7.71 (1H, dd, J = 6.8, 2.1, H-2'''), 6.91 (1H, dd, J = 6.8, 2.1, H-3'''),  6.87 (1H, d, J = 7.5 Hz, H-5'), 5.23 (1H, s, H-1''), 3.95 (3H, s, 4'-OCH_3_), 3.85 (3H, s, 4'''-OCH_3_), 3.82 (3H, s, 3-OCH_3_), 3.74 (3H, s, 6-OCH_3_), 3.52 (2H, t, H-4'') ( 2.63 (2H, t, H-3''); ^13^C NMR (125 MHz, DMSO-d6): δ (ppm) 179.18 (C-4), 159.18 (C-4'''), 157.24 (C-2), 153.61 (C-7), 152.15 (C-9), 150.05 (C-5), 149.14 (C-4'), 147.36 (C-3'), 139.19 (C-3), 136.22 (C-1''') 133.81 (C-6), 128.95 (C-2'''), 123.11 (C-1'), 120.63 (C-6'), 115.21 (C-3''') 114.74 (C-2'), 112.97 (C-8), 112.25 (C-5'), 103.36 (C-10), 71.25 (C-1''), 60.03 (C-4'') 61.58 (6-OCH_3_), 59.72 (3-OCH_3_), 56.19 (4'-OCH_3_), 55.61 (4'''-OCH_3_), 52.86 (C-3''). HREIMS (m/z): Calcd for C₃₀H₃₁NO₁₀ [M]⁺ 565.1948; found 565.1885.

**CA4**


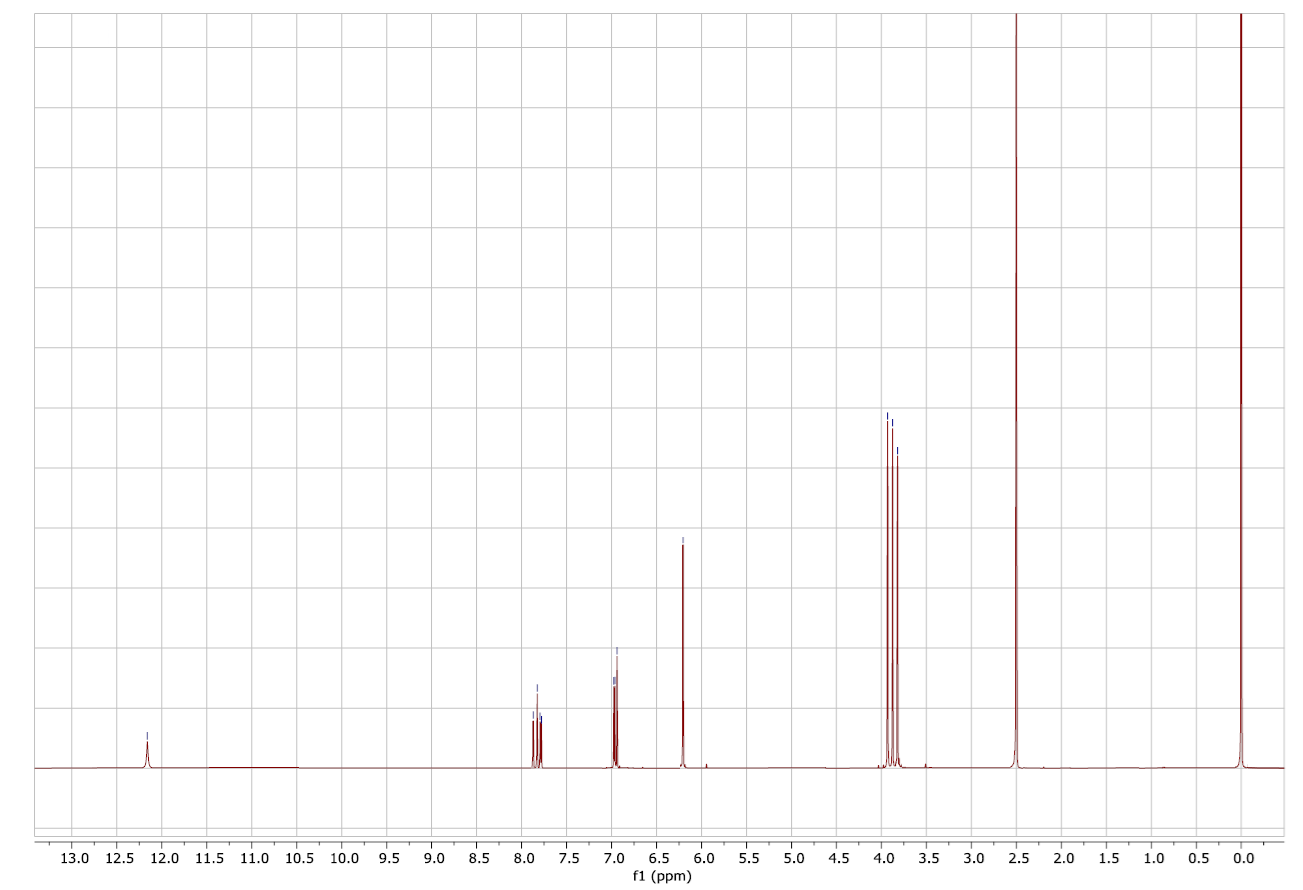


^1^H NMR spectrum of centaureidin in DMSO-d_6_ (500 MHz)


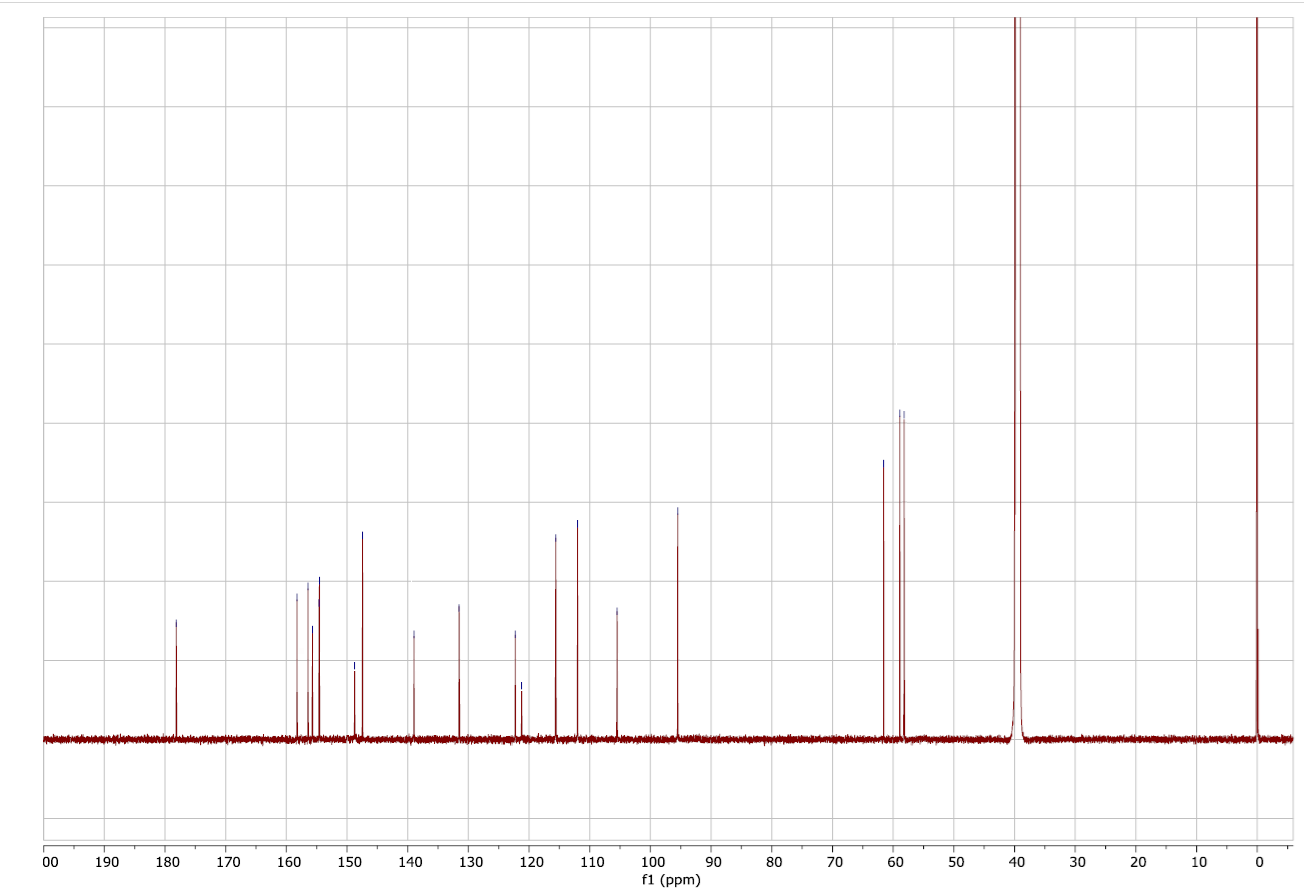


^13^C NMR spectrum of centaureidin in DMSO-d_6_ (125 MHz)


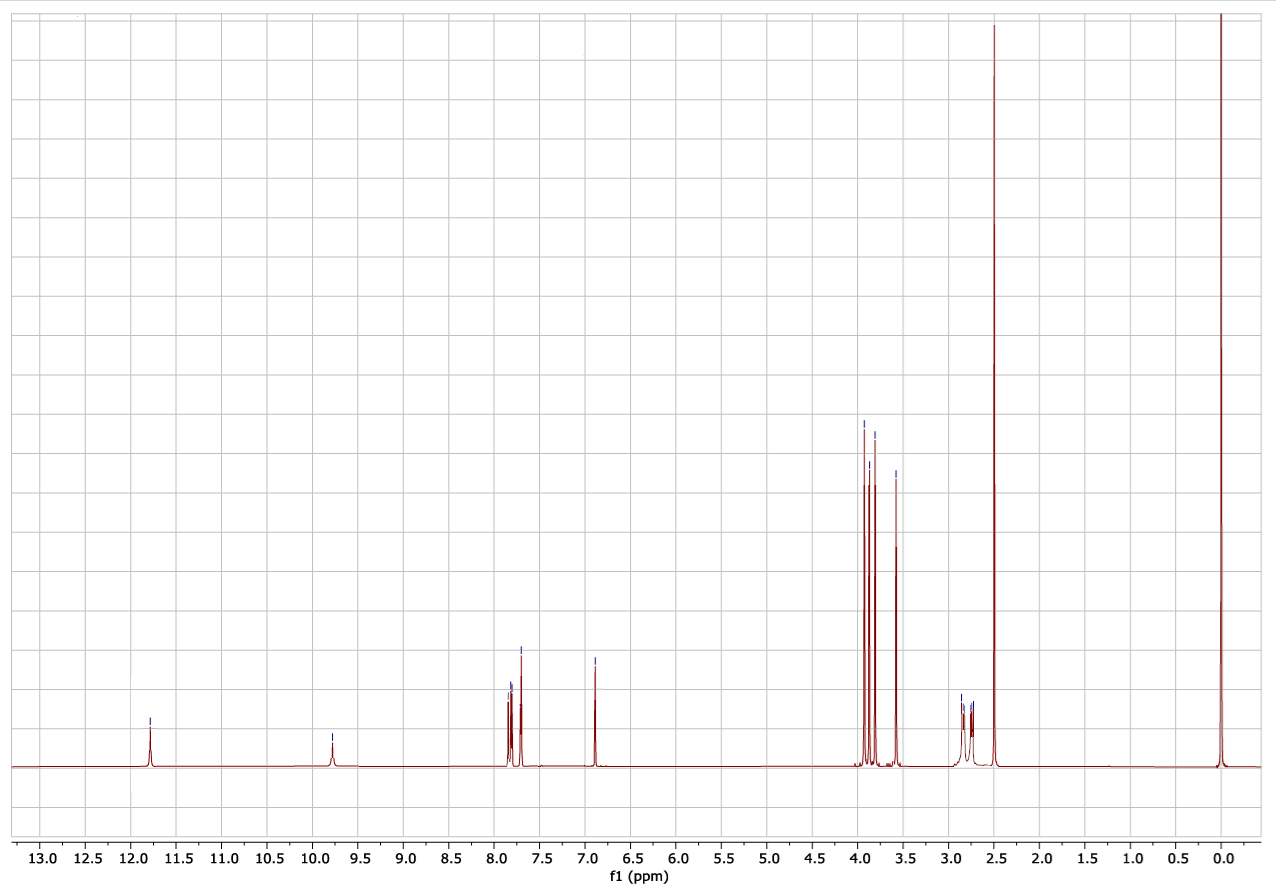


^1^H NMR spectrum of CA1 in DMSO-d_6_ (500 MHz)


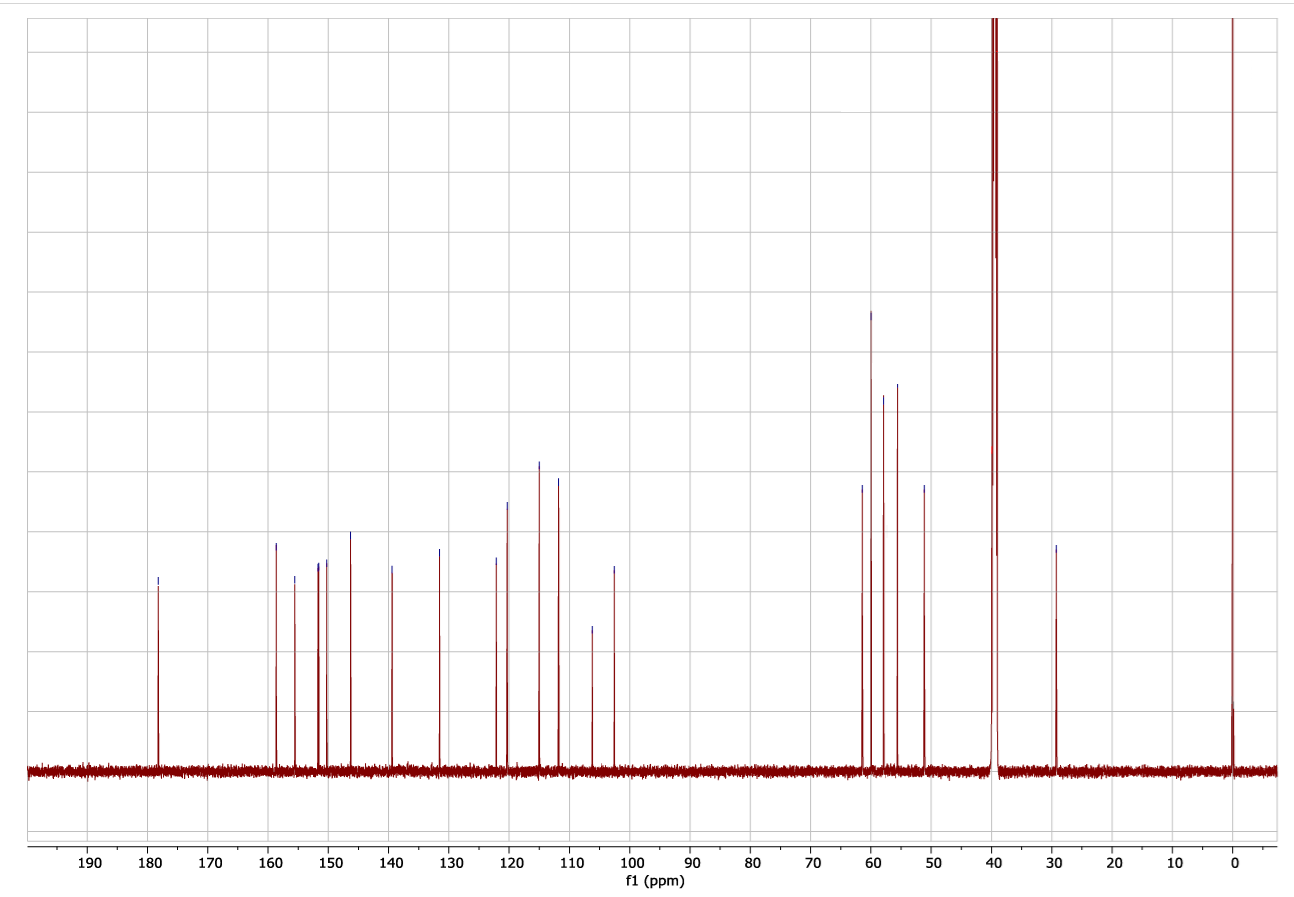


^13^C NMR spectrum of CA1 in DMSO-d_6_ (125 MHz)


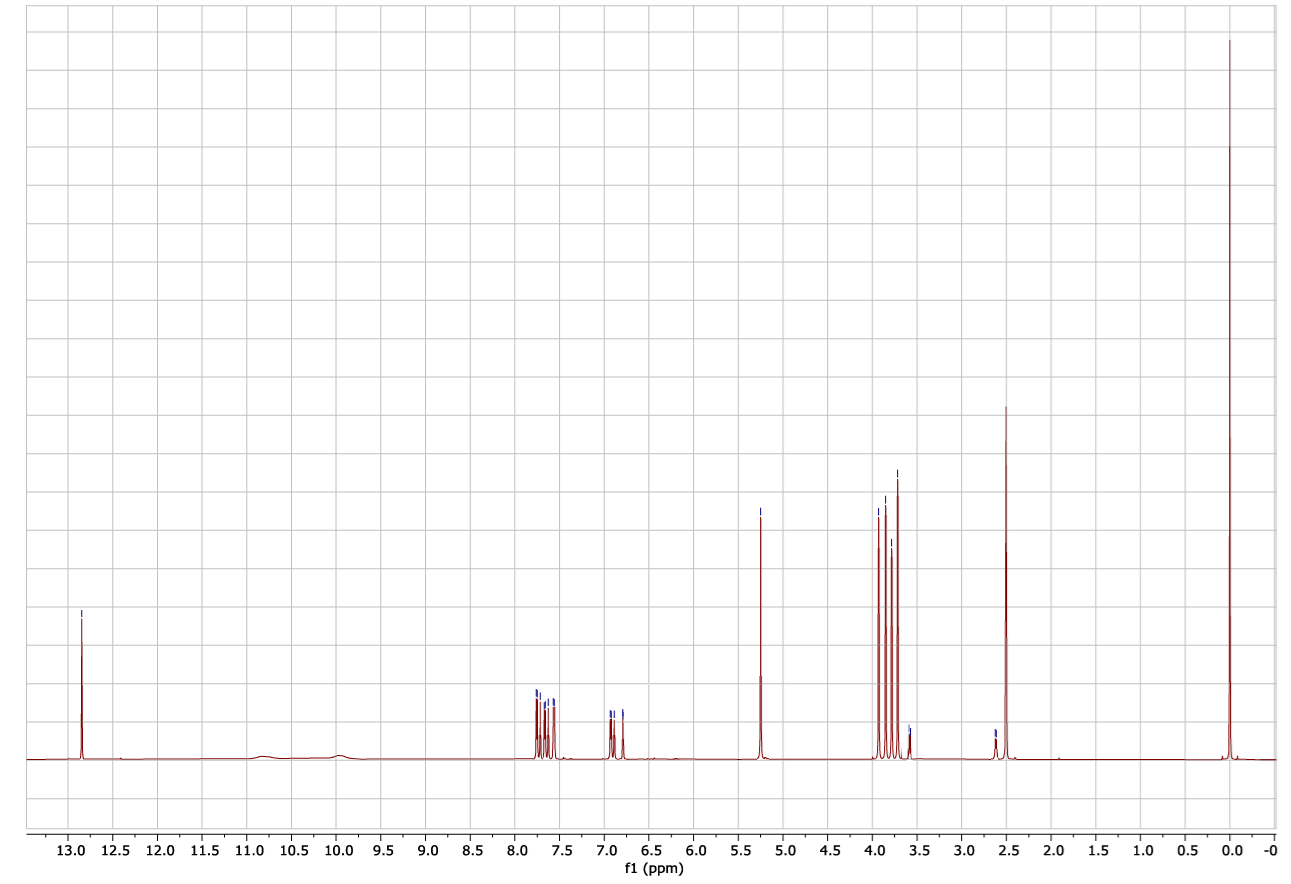


^1^H NMR spectrum of CA4 in DMSO-d_6_ (500 MHz)


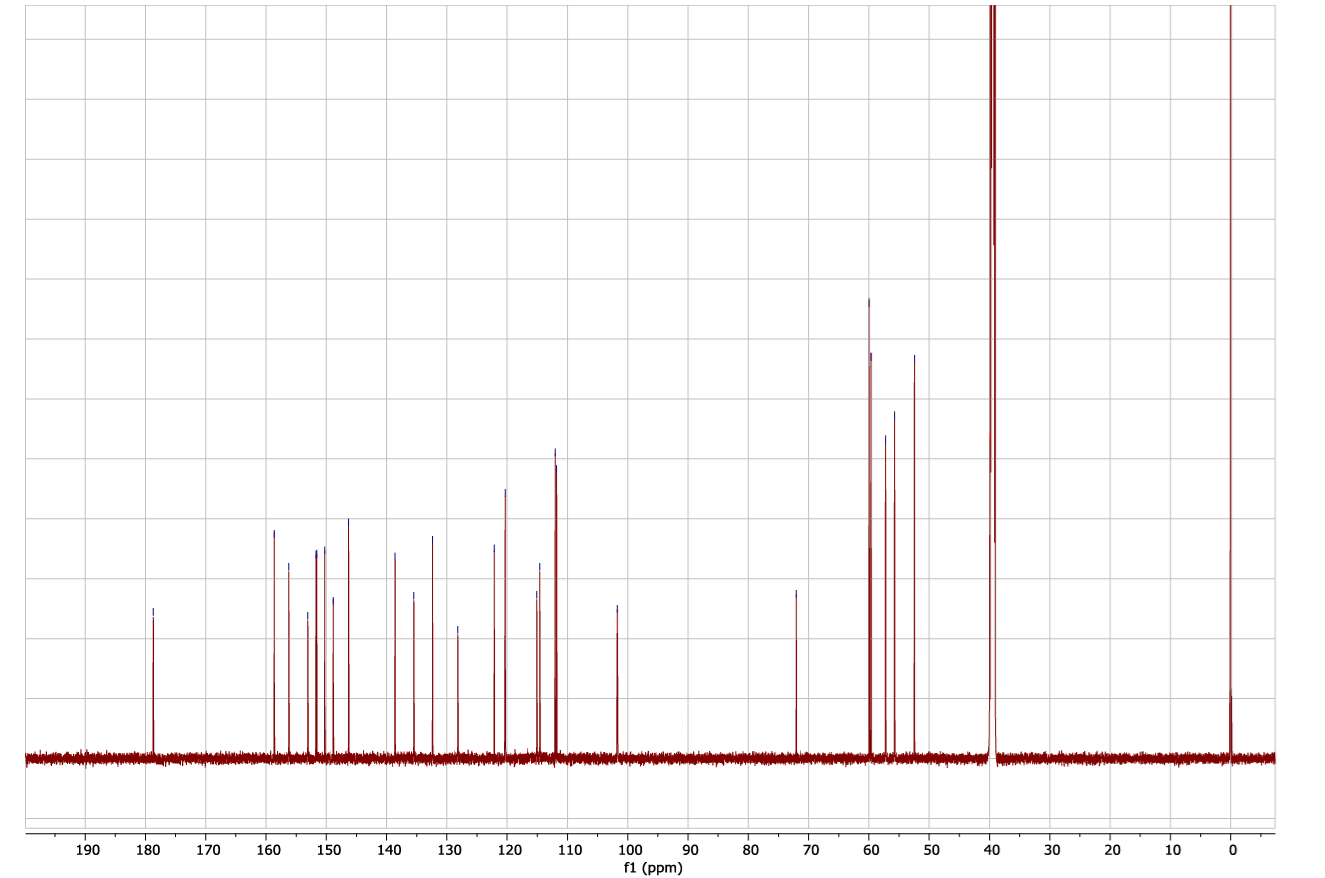


^13^C NMR spectrum of CA4 in DMSO-d_6_ (125 MHz)

**Spectral characterization of investigated compounds**

The Mannich reaction was employed to prepare new compounds by combining a flavonoid, a carbonyl compound (an aldehyde), and a secondary amine. Centaureidin was combined with aldehyde and a heterocyclic secondary amine to afford tetra-substituted ring A of the O-methylated flavonol (Fig. 2). The structures of centaureidin, CA1, and CA4 were elucidated based on data from NMR spectra (supplementary material). Centaureidin was obtained as a yellow oil, the HREIMS mass spectrum revealed a [M + H] ^+^ ion at *m/z* 360.3124 corresponding to the molecular formula C_18_H_16_O_8_. The ^1^H and ^13^C NMR spectrum of centaureidin, recorded in DMSO-𝑑6, revealed a downfield conjugated carbonyl 𝛿_C_ 178.2 (C-4) and displayed a single aromatic proton at 𝛿_H_ 6.32 indicating a single proton at ring A of the flavonoid moiety. The trimethoxylated flavonoid pattern was estimated from the three singlet signals at 𝛿_H_ 3.95, 3.82 and 3.79 ppm, this inference was confirmed by three upfield ^13^C NMR signals at approximately 𝛿_C_ 61, 59 and 57 ppm. The ^1^H NMR profile displayed three aromatic signals at 𝛿_H_ 7.66 (1H, d, J = 2.6 Hz), 7.72 (1H, dd, J = 7.3, 2.6 Hz) and 6.91 (1H, d, J = 7.3 Hz), this coupling pattern is diagnosed for a 1,3,4-trisubstituted aromatic ring B of flavonoids, thus these signals are assigned for protons 2', 6' and 5', respectively.

Compounds CA1 exhibited comparable ^1^H and ^13^C NMR spectral profiles for the main flavonoid backbone of centaureidin. Substitution at C-8 was estimated from the shift of the ^13^C NMR signal of C-8 from 𝛿_C_ 95.37 in centaureidin to 𝛿_C_ 106.27 in CA1, and the disappearing the ^1^H NMR singlet signal of H-8. Two upfield triplets were detected at 𝛿_H_ 2.61 and 2.81 ppm and diagnosed for the two asymmetrical protons of the thiomorpholine 4'' and 3'', respectively. Also, the thiomorpholine carbons appeared at 𝛿_C_ 60.38 for C-3'' and 29.65 for C-4''. The bridge protons at C-1'' were elucidated from a singlet proton signal at 𝛿_H_ 3.59 ppm and a ^13^C NMR signal at 𝛿_C_ 51.23 ppm.

Similarly, the ^1^H and ^13^C NMR spectral patterns of CA4 lie in the same direction as that of centaureidin. The shift of the ^13^C NMR signal of C-8 from 𝛿_C_ 95.37 ppm to a more deshielded signal at 𝛿_C_ 112.97 ppm is indicative of substitution at C-8. The appearance of two new doublets of doublets in the aromatic region at 𝛿_H_ 7.71 and 6.91 is a diagnosis for p-disubstituted benzene. This aromatic ring is confirmed by the appearance of additional aromatic ^13^C NMR signals. The 4'''-OMe in the p-methoxy benzene is elucidated from an extra ^1^H NMR signal at 𝛿_H_ 3.85 and 𝛿_C_ 55.61 ppm. Contrary to CA1, the NMR signals of C-1'' (𝛿_H_ 5.23 (s) and 𝛿_C_ 71.25) is more downfield because of the ring current effect. The morpholine ring is elucidated from two triplets at 𝛿_H_ 3.25 and 2.63 ppm and two ^13^C NMR signals at 𝛿_C_ 52.86 and 68.23 ppm.

**Table S1. Molecular docking grid box size and dimensions**

|  | **Grid box size (Å)** | **Grid box dimension (Å)** |
| --- | --- | --- |
| **Caspase-3** | 40 x 40 x 40 | center_x = 35.808, center_y = 31.296, and center_z = 29.251 |
| **EGFR** | 40 x 40 x 40 | center_x = -52.769, center_y = 2.153, and center_z = -27.729 |
| **HER2** | 40 x 50 x 40 | center_x = 18.755, center_y = 20.514, and center_z = 25.918 |
| **VEGFR 2** | 40 x 40 x 40 | center_x = 36.014, center_y = 34.045, and center_z = 13.867 |

**Table S2. Electronic & steric contributions of the new substituents**

| **Analogue** | **Key substituent(s)** | **How the group stabilizes the phenoxy-type radical generated at 7-OH*** | **Notes** |
| --- | --- | --- | --- |
| **CA1** | Thiomorpholine (contains S) | • **High polarizability of S (3p/3d)**: allows dispersion of the unpaired electron through n(S) → σ*_C–O_ hyperconjugation and through‐bond π-delocalization. • **σ-donor / π-acceptor balance** lowers the spin density on O_7_, reducing the localization penalty. • **Steric bulk** around C-8 hinders radical–radical coupling, prolonging lifetime of the antioxidant intermediate. | Similar S-assisted stabilization is documented for thioether‐modified flavonoids and for S-containing natural antioxidants. |
| **CA4** | (i) para-OMe group (ii) p-C₆H₄-OMe group | • **+M (mesomeric) donation of OMe** injects electron density into the π-system, stabilizing the phenoxy radical by resonance (O–C=C–OMe conjugation). • **Extended conjugation** across the tethered *p*-methoxyphenyl ring provides an additional sink for the unpaired spin (spin delocalization over 19 π-atoms). • **Steric shielding** (torsion of the arm) again retards bimolecular decay. | The methoxy effect is analogous to the well-known stabilization in 4′-OMe-quercetin derivatives. |

** Computational work and experimental assays identify 7-OH as the preferred H-donor site in all analogues*.

**Table S3. Computational evidence**

| **Descriptor (water PCM, UB3LYP/6-311++G(d,p))** | **Centaureidin (rad)** | **CA1 (rad)** | **CA4 (rad)** |
| --- | --- | --- | --- |
| Spin population on O_7_ | 0.48 | 0.34 | 0.31 |
| Spin population on hetero substituent (S or OMe) | – | S = 0.08 | O_OMe_ = 0.06; aryl = 0.07 |
| ⟨S²⟩ (spin contamination) | 0.756 | 0.754 | 0.753 |
| HOMO energy (Hartree) |  | –0.20159 | –0.20092 |
| HOMO energy (eV) |  | –5.49 eV | –5.47 eV |
| LUMO energy (Hartree) |  | –0.10251 | –0.09927 |
| LUMO energy (eV) |  | –2.79 eV | –2.70 eV |
| HOMO–LUMO gap |  | 2.70 eV | 2.77 eV |

Table S4. Binding energies, polar bonds, and hydrophobic interactions of centaureidin, CA1, and CA4 with caspase-3, EGFR, HER2, and VEGFR2.

|  | **Centaureidin** | | | | **CA1** | | | | **CA4** | | |
| --- | --- | --- | --- | --- | --- | --- | --- | --- | --- | --- | --- |
|  | Binding energy (kcal/mol) | Polar bonding | Hydrophobic interactions | Binding energy (kcal/mol) | | Polar bonding | Hydrophobic interactions | Binding energy (kcal/mol) | | Polar bonding | Hydrophobic interactions |
| **Caspase-3** | -7.0 | Arg20, Gln217 and Phe250 | Trp206, Asn208, Asp211, Trp214, Glu246, and Phe247 | -7.1 | | Arg207 and Phe252 | Trp206, Asn208, Trp214, Phe250, Ser251 and Phe256 | -7.5 | | Ser205 and Arg207 | Ser65, Cys163, Trp206, Asn208, Ser209, Trp214, Ser251, Asp253 and Phe256 |
| **EGFR** | -7.6 | Lys745 and Glu762 | Leu718, Val726, Ala743, Ile744, Met766, Leu788, Thr790, Gly796, Leu844 and Thr854 | -7.6 | | Met793 and Thr854 | Leu718, Val726, Ala743, Lys745, Met766, Leu788, Thr790, Gln791, Leu792, Gly796, Leu844 and Asp855 | -6.9 | | Lys745, Glu762 and Asp855 | Phe723, Asp837, Arg841, Asn842, Gly857, Leu858 and Pro877 |
| **HER2** | -7.2 | Lys753, Leu796 and Arg849 | Ser728, Gly729, Val734, Ala751, Ile752, Leu785, Thr798, Cys805, Asn850, Leu852, Thr862 and Asp863 | -7.1 | | Arg985 | Asp950, Met953, Ile954, Lys957, Glu971, Glu975 and Arg978 | -7.7 | |  | Asn708, Asp769, Tyr772, Val839, Leu869, Asp871, and Ile872 |
| **VEGFR 2** | -7.1 |  | Gly843, Phe845, Gly846, Val848, Lys868, Leu870, Glu878, Ala881, Leu882 and Phe1047 | -7.2 | |  | Gly843, Phe845, Gly846, Lys868, Leu870, Glu878, Ala881, Leu882, Asp1028 and Phe1047 | -7.8 | | Ile1025 and Arg1027 | Glu885, Leu889, Ile892, Val899, Cys1024, His1026, Asp1028, Ile1044, Cys1045 and Asp1046 |


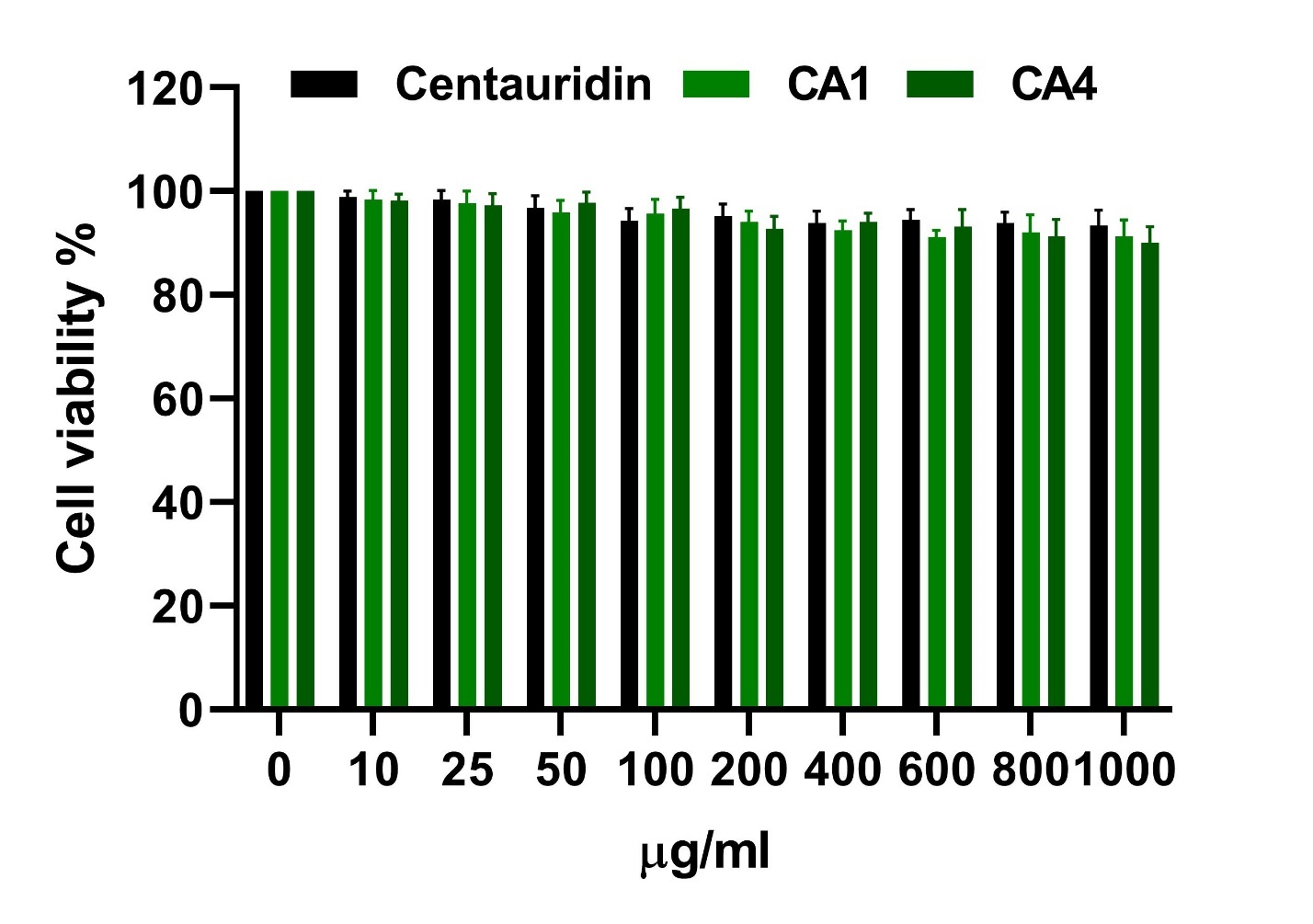


Figure S1. Effect of centaureidin, CA1, and CA4 on HK-2 cell viability.
